# Cellular and molecular insight into the inhibition of primary root growth of Arabidopsis induced by peptaibols, a class of linear peptide antibiotics mainly produced by *Trichoderma* spp.

**DOI:** 10.1093/jxb/erw023

**Published:** 2016-02-05

**Authors:** Wei-Ling Shi, Xiu-Lan Chen, Li-Xia Wang, Zhi-Ting Gong, Shuyu Li, Chun-Long Li, Bin-Bin Xie, Wei Zhang, Mei Shi, Chuanyou Li, Yu-Zhong Zhang, Xiao-Yan Song

**Affiliations:** ^1^State Key Laboratory of Microbial Technology, Marine Biotechnology Research, Shandong University, Jinan 250100, China; ^2^State Key Laboratory of Plant Genomics, National Centre for Plant Gene Research (Beijing), Institute of Genetics and Developmental Biology, Chinese Academy of Sciences, Beijing 100101, China; ^3^Key Laboratory of Plant Cell Engineering and Germplasm Innovation, Ministry of Education, School of Life Science, Shandong University, Jinan 250100, China

**Keywords:** *Arabidopsis thaliana*, auxin, GORK, peptaibols, *Trichoderma*, Trichokonin VI.

## Abstract

*Trichoderma* spp. are well known biocontrol agents that produce a variety of antibiotics. Peptaibols are a class of linear peptide antibiotics mainly produced by *Trichoderma*. Alamethicin, the most studied peptaibol, is reported as toxic to plants at certain concentrations, while the mechanisms involved are unclear. We illustrated the toxic mechanisms of peptaibols by studying the growth-inhibitory effect of Trichokonin VI (TK VI), a peptaibol from *Trichoderma longibrachiatum* SMF2, on *Arabidopsis* primary roots. TK VI inhibited root growth by suppressing cell division and cell elongation, and disrupting root stem cell niche maintenance. TK VI increased auxin content and disrupted auxin response gradients in root tips. Further, we screened the *Arabidopsis* TK VI-resistant mutant *tkr1*. *tkr1* harbors a point mutation in *GORK*, which encodes gated outwardly rectifying K^+^ channel proteins. This mutation alleviated TK VI-induced suppression of K^+^ efflux in roots, thereby stabilizing the auxin gradient. The *tkr1* mutant also resisted the phytotoxicity of alamethicin. Our results indicate that GORK channels play a key role in peptaibol–plant interaction and that there is an inter-relationship between GORK channels and maintenance of auxin homeostasis. The cellular and molecular insight into the peptaibol-induced inhibition of plant root growth advances our understanding of *Trichoderma*–plant interactions.

## Highlight

GORK channels play an important role in the disruption of auxin homeostasis in Arabidopsis root tip and the subsequent inhibition of root growth caused by Trichokonin VI from *Trichoderma*.

## Introduction

*Trichoderma* spp. have long been recognized as agents that can be utilized for the control of plant disease (Benítez *et al.*, 2004). Some *Trichoderma* spp. can produce auxins or volatiles to promote plant growth or protect plants from biotic and abiotic stresses (Contreras-Cornejo *et al.*, 2009, 2014a, b; Hung *et al.*, 2013). The presence of diverse antibiotics correlates well with the biocontrol abilities of *Trichoderma* (Lin *et al.*, 1994; Wilhite *et al.*, 1994) and provides a rich resource for screening new pesticides. In a recent review, the authors emphasized the potential of applications of specific secondary metabolites of *Trichoderma* for the control of phytopathogens as substitutes for whole-organism formulations (Keswani *et al.*, 2014). However, many *Trichoderma* antibiotics are reported to be phytotoxic (Haraguchi *et al.*, 1996; Marfori *et al.*, 2003; Keswani *et al.*, 2014). It was also found that some *Trichoderma* antibiotics, such as harzianic acid and harzianolide, promote plant growth and/or induce plant resistance at low concentrations, but inhibit plant growth at relatively high concentrations (Vinale *et al.*, 2009; Cai *et al.*, 2013). To promote the use of *Trichoderma* and *Trichoderma* antibiotics, a detailed understanding of the phytotoxicity of these antibiotics is necessary.

Peptaibols, a class of linear peptide antibiotics, are mainly produced by *Trichoderma*. Peptaibols are made up of 5–20 amino acid residues, which are synthesized by non-ribosomal peptide synthetases (NRPSs) (Szekeres *et al.*, 2005). Peptaibols can be divided into three groups, which are distinguished by the chain lengths of the amino acid sequences. These groups are as follows: long-sequence peptaibols with 18–20 amino acid residues; short-sequence peptaibols with 11–16 residues; and lipopeptaibols with 6 or 10 residues (Daniel and Filho, 2007). The amphipathic nature of peptaibols allows many of them to form voltage-dependent ion channels in lipid membranes, which is the likely basis of their biological activities (Béven *et al.*, 1999; Chugh and Wallace, 2001), such as antimicrobial activity, antitumor activity, and the ability to elicit plant defense (Szekeres *et al.*, 2005). Alamethicin, the most extensively studied long-sequence peptaibol, is well known for its antimicrobial activity and the ability to induce plant resistance (Leitgeb *et al.*, 2007; Kredics *et al.*, 2013). However, alamethicin can also be toxic to plants. Alamethicin causes lesions on *Arabidopsis* leaves at 5–30 μM (Rippa *et al.*, 2010) and induces rRNA cleavage-associated rapid death in *Arabidopsis* at 50 μM (Rippa *et al.*, 2007). Alamethicin also permeabilizes the plasma membrane and the mitochondria in tobacco suspension cells and induces cell death at ~22 μM (Matic *et al.*, 2005). The mechanisms involved are not yet fully understood.

Post-embryonic root growth in higher plants is maintained by the root meristem. Within the root meristem, the mitotically inactive quiescent center (QC) cells and the surrounding stem cells constitute a specialized microenvironment known as the root stem cell niche, which serves as the source of all differentiated cell types during root development (Dello Ioio *et al.*, 2008; Dinneny and Benfey, 2008). The phytohormone auxin plays a crucial role in root patterning. It is generally believed that local auxin biosynthesis and polar auxin transport, which depends on the PIN-FORMED (PIN) family of auxin efflux facilitators, function together to establish a state of auxin homeostasis that is required for root meristem patterning and maintenance (Jiang and Feldman, 2003; Blilou *et al.*, 2005; Zhao, 2010).

*Trichoderma longibrachiatum* SMF2 (hereafter SMF2) is a biocontrol fungus that produces several long-sequence peptaibols known as Trichokonins (TKs), which have broad-spectrum antimicrobial activity (Song *et al.*, 2006; Shi *et al.*, 2012). TK VI is the main component of the TKs produced by SMF2 (Song *et al.*, 2007). In addition to being an antibiotic, TK VI has other biological functions, including the elicitation of systemic resistance in tobacco and Chinese cabbage (Luo *et al.*, 2010; Li *et al.*, 2014) and the induction of programmed cell death in tumor cells (Shi *et al.*, 2010). However, the effect of TK VI on plant growth has not yet been studied.

In this study, we investigated the cellular and molecular mechanisms of TK VI-induced inhibition of primary root growth in *Arabidopsis thaliana*. TK VI inhibited both cell division and cell elongation in the primary root, and disturbed the maintenance of the root stem cell niche. TK VI also increased the auxin content and disrupted the auxin response gradients in the root tip by modulating local auxin biosynthesis and polar auxin transport. We screened the dominant mutant *tkr1*, which resisted TK VI toxicity, to further elucidate the molecular mechanisms involved. Map-based cloning revealed a mutation in the *GORK* gene, which encodes a gated outwardly rectifying K^+^ channel protein. Using the non-invasive micro-test technique (NMT) and patch-clamp whole-cell recordings, we found that K^+^ efflux was suppressed by TK VI in the wild type and this suppression was much alleviated in *tkr1*, enabling *tkr1* to maintain K^+^ and auxin homeostasis better than the wild type when TK VI was supplied. Moreover, we found that *tkr1* was also resistant to root growth inhibition caused by alamethicin. Our results revealed the cellular and molecular mechanisms involved in the peptaibol-induced inhibition of plant root growth, which advances our understanding of the interactions between *Trichoderma* and plants.

## Materials and methods

### Plant materials and growth conditions

The *A. thaliana* ecotypes Columbia-0 (Col-0) and Landsberg *erecta* (L*er*) were used. Some of the plant materials used in this study were previously described *CYCB1;1*_*pro*_ *:GFP* (Zheng *et al.*, 2011); QC25 (Sabatini *et al.*, 1999); *DR5*_*pro*_ *:GUS* (Ulmasov *et al.*, 1997); *IAA2*_*pro*_ *:GUS* (Swarup *et al.*, 2007); *PIN1*_*pro*_ *:GUS*, *PIN2*_*pro*_ *:GUS*, *PIN3*_*pro*_ *:GUS*, and *PIN7*_*pro*_ *:GUS* (Vieten *et al.*, 2005); *ASA1*_*pro*_ *:GUS* and *ASB1*_*pro*_ *:GUS* (Stepanova *et al.*, 2005); *axr1-1* (Lincoln *et al.*, 1990); *pin1-5* (Sohlberg *et al.*, 2006); *pin2 pin3 pin4*, *pin3 pin4 pin7*, and *pin2 pin3 pin7* (Blilou *et al.*, 2005); *asa1-1* (Sun *et al.*, 2009); *PLT1*_*pro*_ *:CFP*, *PLT1*_*pro*_ *:PLT1:YFP*, *PLT2*_*pro*_ *:CFP*, and *PLT2*_*pro*_ *:PLT2:YFP* (Kornet and Scheres, 2009); *SHR*_*pro*_ *:SHR:GFP* (Nakajima *et al.*, 2001); and *SCR*_*pro*_ *:GFP* (Helariutta *et al.*, 2000). Salk_082258C seeds were obtained from the *A. thaliana* Biological Resource Center.

Seeds were surface-sterilized for 15min in 10% (v/v) commercial bleach, washed five times with sterile water, and plated on half-strength Murashige and Skoog (MS) medium with 1% (w/v) sucrose and 0.8% (w/v) agar. The seeds were stratified at 4 °C for 2 d in the dark and then transferred to a phytotron set at 22 °C with a 16h light/8h dark photoperiod (with a light intensity of 120 μmol photons m^–2^ s^–1^) in vertically or horizontally oriented Petri dishes.

### Purification and preparation of TK VI

TK VI was prepared from solid-state fermented SMF2 using a previously described method (Song *et al.*, 2007). Purified TK VI powder (purity >95%) was dissolved in methanol to obtain a 5mM stock solution for further use.

### Phenotypic analysis, statistics, and microscopy

The representative seedlings were transplanted to a new Petri dish to be photographed, and root lengths were measured using Image J. The size of the root meristem was assessed as the number of cells between the QC and the first elongating cell in the cortex cell file (Dello Ioio *et al.*, 2007). Whole seedlings or different tissues were cleared in HCG solution (chloroacetaldehyde:water:glycerol=8:3:1) for several minutes before microscopic analysis. Microscopy was performed using a Leica Microsystems DM5000B microscope and DFC490 charge-coupled device (CCD) camera. Statistical significance was evaluated using Student’s *t*-test. The presented data are mean values of at least three biological repeats with SD.

For Lugol staining of the roots, tissues were incubated in Lugol solution (Sigma-Aldrich) for 3–5min, washed in water once, and mounted in HCG solution for microscopic analysis (Chen *et al.*, 2011). Histochemical staining for β-glucuronidase (GUS) activity in transgenic plants was performed as previously described (Jefferson *et al.*, 1987). Confocal imaging was obtained using a Leica TCS SP5 confocal laser-scanning microscope. Seedlings were stained with 10mg ml^–1^ propidium iodide for 5min and washed once in water. Fluorescence was quantified using the LAS AF Lite program. Approximately 10 seedlings per image were examined. At least three independent experiments were performed to obtain consistent and statistically significant results.

### Gene expression analysis

For quantitative reverse transcription–PCR (qRT–PCR) analysis, whole seedlings or root tip tissues were harvested and frozen in liquid nitrogen for RNA extraction. RNA was extracted using the RNeasy kit (Qiagen). First-strand cDNA was synthesized from 2 μg of total RNA using Superscript III reverse transcriptase (Invitrogen) and oligo(dT) primers. qRT–PCRs were performed using a cycler apparatus (Bio-Rad) and the RealMasterMix kit (SYBR Green, Tiangen) according to the manufacturer’s instructions. The expression levels of the target genes were normalized to those of *ACTIN2*. Statistical significance was evaluated by Student’s *t*-test. The primers used to quantify gene expression levels are listed in Supplementary Table S1 at *JXB* online.

### Free IAA measurement

Five-day-old Col-0 seedlings were transplanted to half-strength MS medium without or with 5 μM TK VI for 3h. Then, 2mm root tips were collected for indole acetic acid (IAA) measurements. Approximately 150mg (fresh weight) of root tips was used for IAA extraction and measurement, which followed a published method (Kojima *et al.*, 2009) with minor modifications (Zhou *et al.*, 2010).

### Polar auxin transport assay

Five-day-old seedlings grown vertically on plates were transferred to medium without or with 5 μM TK VI for 24h. Seedlings were then transferred to half-strength MS medium, and 1cm of the apical ends of the roots was removed for polar auxin transport assays as previously described (Lewis and Muday, 2009) with minor modifications (Qi *et al.*, 2012).

### Mutant screening and map-based cloning of the *GORK* gene

The seeds of an ethylmethane sulfonate (EMS)-mutagenized population of *Arabidopsis* ecotype Col-0 were grown vertically on medium supplied with 5 μM TK VI and screened for seedlings with longer roots compared with those of the wild type. Seedlings with longer primary roots were transplanted to soil so that seeds could be harvested for further verification. One mutant, named *tkr1*, displayed consistent TK VI resistance. The original *tkr1* mutant was backcrossed to Col-0 for three generations, and the resulting homozygous progeny were used in this study.

For mapping analysis, the *tkr1* mutant in the Col-0 background was crossed with the polymorphic ecotype L*er*. The resulting F_1_ plants were self-pollinated to yield an F_2_ population segregating for *tkr1* mutant phenotypes. A total of 1200 individual F_2_ plants were selected for *GORK* chromosomal mapping.

To generate the *DR5*_*pro*_ *:GUS* reporter line in the *tkr1* mutant background, homozygous *tkr1* plants were crossed with a transgenic line harboring the *DR5*_*pro*_ *:GUS* construct to produce an F_2_ population. Plants that were homozygous for the *tkr1* mutation and the *DR5*_*pro*_ *:GUS* construct were identified from the F_2_ population and analyzed in the next generation. In each analysis, parental transgenic lines were used for comparisons with corresponding mutants.

### Plasmid construction and plant transformation

The *GORK* promoter region was PCR amplified using the primers 5'-CGC*GGATCC*ATTGTTATAGATCACTTTAAG-3' (*Bam*HI) and 5'-CCG*GAATTC*GTTTTCAAGAATCGGTTAAATGAAA TC-3' (*Eco*RI). The PCR product was cloned into the *Bam*HI/*Eco*RI sites of the binary vector pCAMBIA1391-Z (CAMBIA), resulting in a transcriptional fusion of the *GORK* promoter and the GUS-coding region.

Full-length GORK-coding sequences were amplified from wild-type and *tkr1* genomes using Gateway-compatible primers. The PCR products were cloned using pENTR Directional TOPO cloning kits (Invitrogen) and then recombined with the binary vector pGWB2 (Nakagawa *et al.*, 2007) to generate the *35S*_*pro*_ *:GORK* and *35S*_*pro*_ *:tkr1* constructs. The primers used were 5'-CACCATGGGACGTCTCCGGAGA-3' and 5'-TTATGTTTGATCAGTAGTATCACTG-3'.

The *GORK* promoter region was amplified using the primers 5'-AA*CTGCAG*ATTGTTATAGATCACTTTAAG-3' (*Pst*I) and 5'-CGG*GGTACC*GTTTTCAAGAATCGGTTAAATGAAATC-3' (*Kpn*I) for cloning into the *Pst*I/*Kpn*I sites of vector pCAMBIA1300 (CAMBIA). The full-length GORK-coding sequence in *tkr1* was amplified using the primers 5'-CGG*GGTACC*ATG GGACGTCTCCGGAGACGGCAAG-3' (*Kpn*I) and 5'-CGG *GGTACC*TGTTTGATCAGTAGTATCACTG-3' (*Kpn*I) for cloning into the *Kpn*I site of the former construct to generate the *GORK*_*pro*_ *:tkr1* construct. The orientation of the insertion was confirmed by sequencing.

The above constructs were then transformed into the *Agrobacterium tumefaciens* strain GV3101 (pMP90), which was used for the transformation of *Arabidopsis* plants by vacuum infiltration (Bechtold and Pelletier, 1998). Transformants were selected based on their resistance to hygromycin (Roche).

### Measurement of net K^+^ flux using NMT

Transient K^+^ flux was measured using a BIO-IM Series NMT system (YoungerUSA) at the Xuyue Beijing NMT Research Service Center as previously described (Vincent *et al.*, 2005). Five-day-old seedlings were incubated in measuring solution (0.1mM CaCl_2_, 0.1mM KCl, and 0.3mM MES, pH 5.8) for 15min to equilibrate before the K^+^ ion flux measurements were recorded for 10–15min. Then, 12 µM TK VI was added to a final concentration of 3 µM. The K^+^ ion flux recordings were restarted and continued for 14min. The data measured during the first 2min after the TK VI shock were discarded because of diffusion effects from the addition of the stock solution. Net fluxes were calculated using JCal V3.2.2 (http://youngerusa.com or http://ifluxes.com/jcal).

### Patch-clamp whole-cell recordings of protoplasts isolated from the root elongation zone epidermis

The standard and enzyme solutions for protoplasts isolation were the same as those previously described (Xu *et al.*, 2006). Root tips (2–3mm) were isolated and incubated in enzyme solution at 23 ºC for 30min. It was confirmed by direct observation that protoplasts were released solely from the elongation zone epidermis (Demidchik *et al.*, 2003). Protoplasts were filtered through a 50 µm nylon mesh and washed twice with standard solution. The bath and pipette solutions used for whole-cell recordings were the same as those previously described (Ivashikina *et al.*, 2001).

Whole-cell currents were recorded 10min after whole-cell configuration was achieved using an Axopatch-200B amplifier (Axon Instruments, USA). For the recordings, the holding potential was set as –60 mV; the test voltage steps were from –120 mV to 100 mV, with +20 mV increments and a 5s duration for each test voltage. For TK VI-treated samples, 3 μM TK VI was added to the bath solution immediately after the formation of the whole-cell configuration. Currents were recorded after 10min. The software used for data recording and analysis was pCLAMP 10.2. The Student’s *t*-test was used for the comparison of current magnitudes. Results with *P*≤0.001 were considered to be significantly different.

## Results

### TKs produced by *Trichoderma longibranchiatum* SMF2 inhibit the growth of Arabidopsis seedlings

**Fig. 1.**
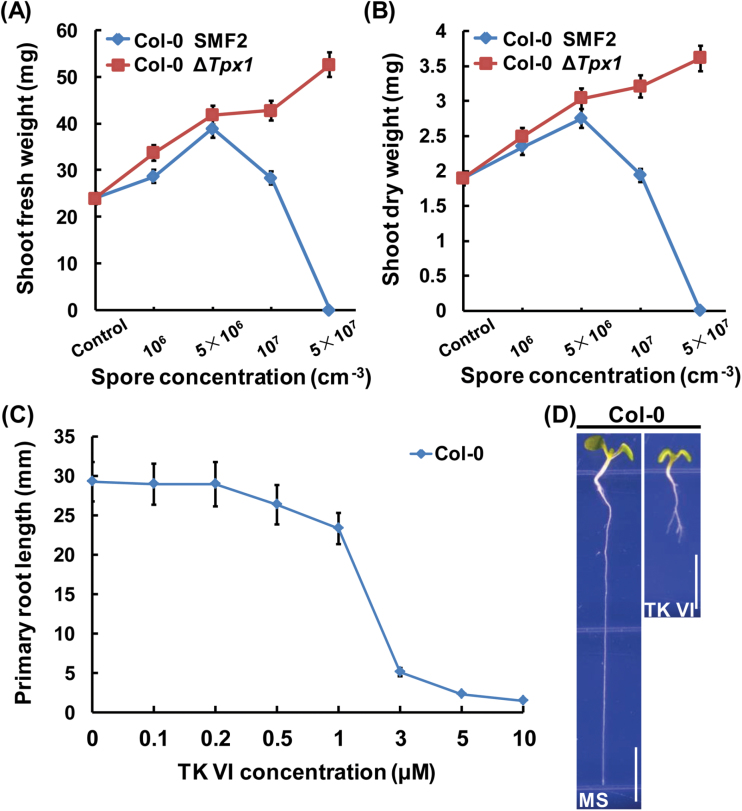
Growth inhibition of Col-0 seedlings by TKs produced by SMF2. (A, B) Statistical analysis of shoot fresh weight (A) and shoot dry weight (B) of Col-0 seedlings grown in soil without (Control) or with different concentrations of spores from wild-type SMF2 and Δ*Tpx1.* Δ*Tpx1* is a mutant of SMF2 that does not produce TKs. Eight-day-old Col-0 seedlings were transplanted to soil without or with the indicated concentrations of spores from wild-type SMF2 and Δ*Tpx1*. Plants were grown for an additional 2 weeks before their shoots were removed and weighed immediately (A) or weighed after drying at 65 °C for 3 d (B). Data shown are representative of at least three independent experiments. Each point represents the mean of 36 seedlings, and the error bars represent the SD of triplicate measurements. (C) Primary root length of 6 DAG (days after germination) Col-0 seedlings grown on medium with 0–10 μM TK VI. Data shown are averages with the SD (*n*>20). (D) Col-0 seedlings grown on medium without (MS) or with 5 μM TK VI at 6 DAG. Scale bars=5mm. (This figure is available in colour at *JXB* online.)

To study the effect of TKs on plant growth in SMF2–plant interactions, we grew *Arabidopsis* seedlings in soil supplied with different concentrations of spores from the wild-type SMF2 strain and the Δ*Tpx1* strain. Δ*Tpx1* is a mutant of SMF2 that does not produce TKs (Supplementary Fig. S1). Low concentrations (≤5×10^6^ cm^–3^) of wild-type SMF2 spores supplied in the soil promoted the growth of *Arabidopsis* seedlings. However, high concentrations (≥1×10^7^ cm^–3^) of wild-type SMF2 spores counteracted this growth promotion effect or inhibited the growth of the *Arabidopsis* seedlings. Treatment with spores of the Δ*Tpx1* strain did not show any negative effect, but produced a dose-dependent promotion effect on the growth of the *Arabidopsis* seedlings (Fig. 1A, B; Supplementary Fig. S2). These results suggest that SMF2 TKs can counteract the plant growth promotion effect of SMF2 or even inhibit plant growth.

TK VI is the main component of the TKs produced by SMF2. To confirm further that TKs affect plant growth, we studied the effect of TK VI on the growth of *Arabidopsis* seedlings. We measured the primary root length of *Arabidopsis* seedlings by germinating seeds in medium containing different concentrations of TK VI. TK VI had no obvious effect on primary root growth at concentrations <0.5 µM, but inhibited primary root growth in a dose-dependent manner at concentrations within a range of 0.5–10 µM, with a reduction of 92% of root growth by 5 μM TK VI (Fig. 1C, D) The biomass of *Arabidopsis* shoots and roots was consistently and significantly reduced by 5 µM TK VI (Supplementary Fig. S3).

### TK VI-induced inhibition of primary root growth involves reductions in both root meristem activity and root elongation

**Fig. 2.**
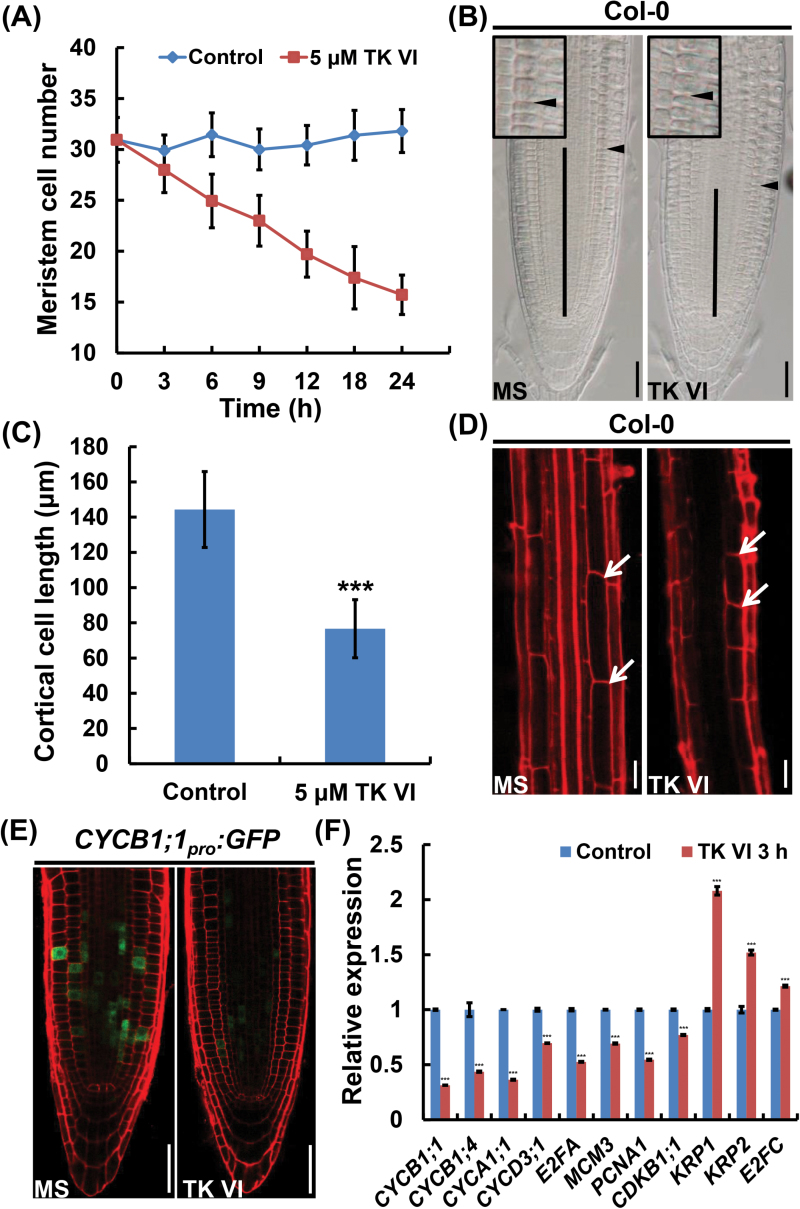
TK VI-induced inhibition of cell proliferation and cell elongation in the Col-0 root tip. (A) Time course of TK VI-induced reductions in root meristem size in Col-0 seedlings at 5 DAG. Data shown are averages with the SD (*n*=20). (B) Root meristems of 5 DAG Col-0 seedlings transplanted to medium without (MS) or with 5 μM TK VI for 12h. The meristem is marked with a vertical black line. The boundary between the meristem zone and the elongation zone is marked with a black arrowhead. The black box is an enlarged image of the boundary between the meristem zone and the elongation zone. (C) Cortical cell length in the differentiation zone of 5 DAG Col-0 seedlings grown on medium without (Control) or with 5 μM TK VI. Data shown are averages with the SD (*n*=20). The asterisks denote Student’s *t*-test significance compared with untreated plants: ***P<0.001. (D) Differentiation zones of seedlings mentioned in (C). The white arrowheads indicate the length of a single cortical cell. (E) TK VI-induced reduction in *CYCB1;1*_*pro*_ *:GFP* expression in Col-0. Six-day-old seedlings were transferred to medium without (MS) or with 5 μM TK VI for 3h before GFP fluorescence was monitored. (F) The effect of TK VI on the expression of cell cycle-related genes in the Col-0 root tip. Six-day-old Col-0 seedlings were transplanted to medium without (Control) or with 5 μM TK VI for 3h, and the 2mm root tips were harvested for RNA extraction and qRT–PCR analysis. The transcript levels of the indicated genes in Col-0 without TK VI treatment were arbitrarily set to 1. The error bars represent the SD of triplicate reactions. The asterisks denote Student’s t-test significance compared with untreated plants: ***P<0.001. Scale bars=50 μm (B, D, E).

Because root growth depends on both cell division in the meristem zone and cell extension in the elongation region, we investigated TK VI-induced cellular changes in these two zones. As expected, the application of TK VI decreased the meristem size due to a progressive reduction in the number of meristematic cells (Fig. 2A, B). Additionally, shortened cortex cells in the root differentiation zone indicated that cell extension was also inhibited by TK VI in the elongation zone (Fig. 2C, D).

To determine whether the TK VI-induced decrease in the root meristem cell number was a result of a negative effect on cell division, we monitored the expression of the transcriptional fusion reporter *CYCB1;1*_*pro*_ *:GFP*, a marker that indicates the G_2_/M phase of the cell cycle. *CYCB1;1*_*pro*_ *:GFP* was expressed in actively dividing cells of the root meristem, and the application of TK VI markedly reduced the expression range of *CYCB1;1*_*pro*_ *:GFP* (Fig. 2E). In a qRT–PCR assay, TK VI markedly reduced the expression levels of multiple cell cycle-related genes that positively regulate cell division, including *CYCB1;1*, *CYCB1;4*, *CYCA1;1*, *CYCD3;1*, *E2FA*, *MCM3*, *PCNA1*, and *CDKB1;1*, while the expression of three negative regulators of cell division in *Arabidopsis*, *KRP1*, *KRP2*, and *E2FC*, was up-regulated accordingly (Fig. 2F) (Gutierrez, 2009).

### TK VI disrupts the maintenance of the root stem cell niche

**Fig. 3.**
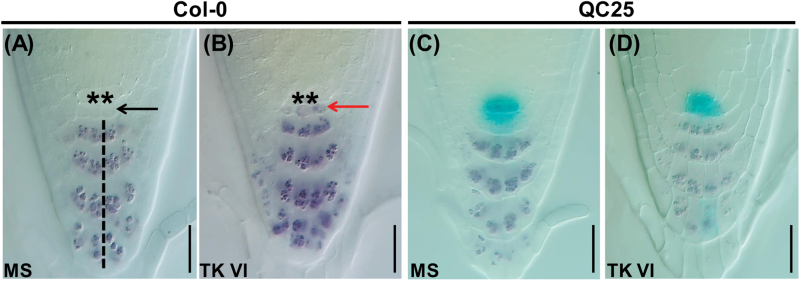
TK VI-induced disruption of the maintenance of the root stem cell niche in Col-0. (A, B) TK VI-induced CSC differentiation in Col-0 as shown by Lugol staining. Six-day-old seedlings were transferred to medium without (MS) (A) or with (B) 5 μM TK VI for 12h before Lugol staining (dark brown). The asterisks denote QC cells. The black dashed line indicates the columella cell layers. The black arrowhead indicates a lack of starch accumulation in non-differentiated CSCs. The red arrowhead shows starch accumulation in TK VI-treated CSCs. (C, D) Disorganized QC cells and differentiated CSCs as shown by Lugol staining of the QC25 marker line. Six-day-old seedlings were transplanted to medium without (MS) (C) or with (D) 5 μM TK VI for 48h before double staining with GUS and Lugol was performed. Scale bars=20 µm (A–D).

Our finding that TK VI alters root meristem activity prompted us to investigate its possible effect on the cellular organization of the QC and its surrounding stem cells. We analyzed the columella stem cells (CSCs), the cell layer immediately below the QC, to assess the possible effect of TK VI on stem cell maintenance. In the Lugol staining assay, untreated CSCs did not show starch granule accumulation (Fig. 3A, black arrow), which were thus distinct from the underlying well-organized columella cells that contained accumulated starch granules (Fig. 3A, black dashed line). After 12h of TK VI treatment, irregular CSCs containing starch granules were clearly observed immediately below QC cells (Fig. 3B, red arrow), indicating that the CSCs were in a state of differentiation. In the GUS and Lugol double-stained QC marker line QC25, aberrant cellular organization in the QC was visible after prolonged treatment with TK VI for 48h, and the accumulation of starch granules in CSCs was much more severe (Fig. 3C, D).

### TK VI increases auxin content and alters auxin response gradients in the root tip

**Fig. 4.**
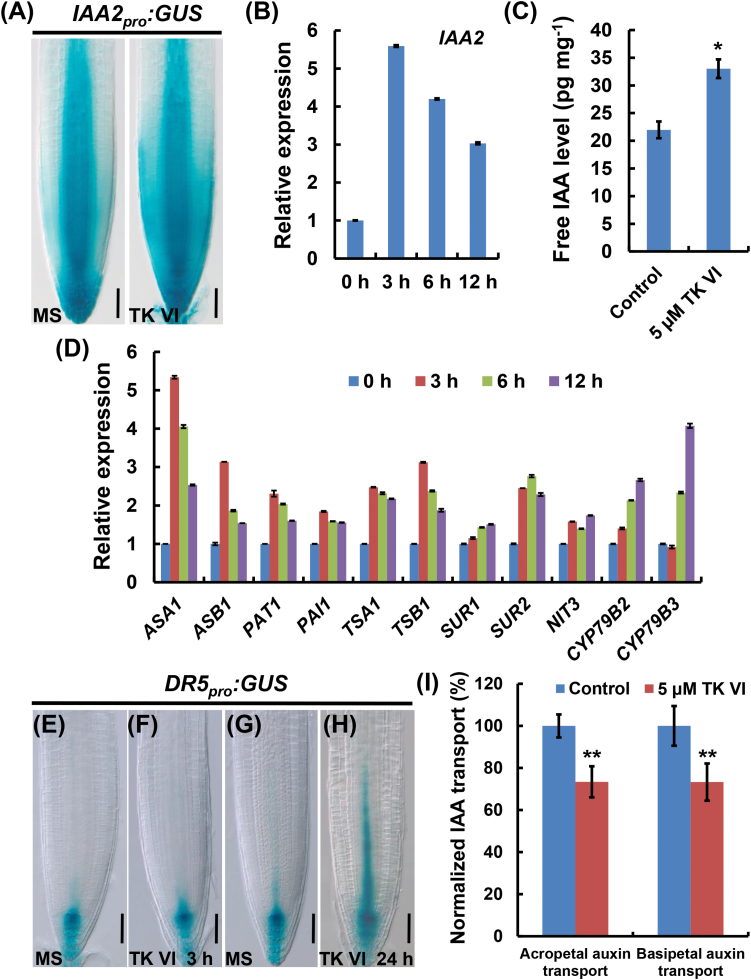
TK VI-induced increase of auxin content and alteration of auxin response gradients in the Col-0 root tip. (A) TK VI-induced enhancement of *IAA2*_*pro*_ *:GUS* expression in the Col-0 root tip. Six-day-old seedlings were transferred to medium without (MS) or with 5 μM TK VI for 12h before GUS staining. (B) Time course of expression of *IAA2* in response to TK VI treatment. (C) Free IAA measurement in Col-0 root tips in response to TK VI treatment. Six-day-old Col-0 seedlings were transplanted to medium without (Control) or with 5 μM TK VI for 3h before the 2mm root tips were harvested. Free IAA levels were then measured. The error bars represent the SD of triplicate measurements. The asterisks denote Student’s *t*-test significance compared with untreated plants: *P<0.05. (D) Time course of expression of auxin biosynthesis-related genes in response to TK VI treatment. For (B) and (D), 6-day-old Col-0 seedlings were treated with 5 μM TK VI for the indicated time periods, and the 2mm root tips were harvested for RNA extraction and qRT–PCR analysis. Transcript levels of the indicated genes in Col-0 without TK VI treatment were arbitrarily set to 1. The error bars represent the SD of triplicate reactions. (E, F, G, H) TK VI-induced disturbance of *DR5*_*pro*_ *:GUS* expression in the Col-0 root tip. Six-day-old seedlings were transferred to medium without (MS) or with 5 μM TK VI for the indicated times before GUS staining. (I) TK VI-induced repression of acropetal and basipetal auxin transport. Six-day-old Col-0 seedlings were transplanted to medium without (Control) or with 5 μM TK VI for 24h before acropetal and basipetal auxin transport assays were performed. The acropetal and basipetal auxin transport levels without TK VI treatment were arbitrarily set to 100%. Data shown are mean values of five biological repeats with the SD. The asterisks denote Student’s *t*-test significance compared with untreated plants: **P<0.01. Scale bars=50 µm (A, E, F, G, H).

Considering the critical role of the phytohormone auxin in root growth and development, the auxin response in *Arabidopsis* root tips upon TK VI treatment was monitored using the auxin-inducible reporter *IAA2*_*pro*_ *:GUS*. Upon TK VI treatment, *IAA2*_*pro*_ *:GUS* was ectopically induced in the root tip (Fig. 4A). This finding, together with the qRT–PCR analysis of *IAA2* expression (Fig. 4B), indicated that auxin was probably accumulated in the root tip. We then measured free IAA in the root tip. As expected, the free IAA level in TK VI-treated root tips was 50% greater than that in untreated root tips (Fig. 4C). Using qRT–PCR, we found that TK VI treatment increased the transcription levels of several auxin biosynthesis-related genes in the root tip (Fig. 4D). Our promoter–GUS assays also indicated that TK VI significantly enhanced the expression of *ASA1* and *ASB1* (Supplementary Fig. S4). Collectively, these data suggest that TK VI induces IAA biosynthesis in the *Arabidopsis* root tip through the transcriptional activation of auxin biosynthetic genes.

The accumulation of IAA in the root tip prompted us to examine the possible effect of TK VI on auxin gradients, which are mainly generated by *PIN* gene-mediated polar auxin transport (Blilou *et al.*, 2005). As shown using the auxin-responsive *DR5*_*pro*_ *:GUS* reporter, auxin is asymmetrically distributed in the root tip, with an apparent high concentration (auxin maximum) in the QC/columella initial region (Fig. 4E, G), and auxin response gradients in the root tip were not altered after TK VI treatment for 3h (Fig. 4F). Accordingly, the expression levels of the auxin efflux transporter genes, *PIN1*, *PIN2*, *PIN3*, and *PIN7*, as indicated by *PIN1*_*pro*_ *:GUS*, *PIN2*_*pro*_ *:GUS*, *PIN3*_*pro*_ *:GUS*, and *PIN7*_*pro*_ *:GUS*, were not visibly affected by 3h TK VI treatment (Supplementary Fig. S5). Instead, *DR5*_*pro*_ *:GUS* expression was induced above the QC/columella initial region in the stele after prolonged treatment with TK VI for 24h (Fig. 4H), indicating the disruption of auxin response gradients in the root tip. Moreover, with TK VI treatment for 24h, the acropetal and basipetal auxin transport rates in the root tip both reduced by ~27% compared with that of the controls (Fig. 4I).

### Isolation and phenotyping of the Arabidopsis TK VI-resistant mutant *tkr1*

**Fig. 5.**
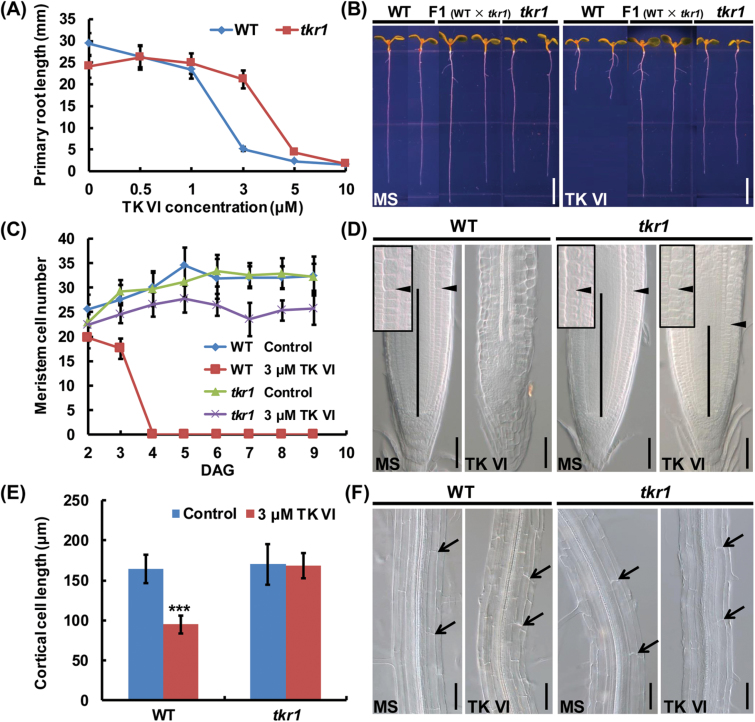
Isolation and phenotyping of the TK VI-resistant mutant *tkr1*. (A) Primary root length of 6 DAG wild-type (WT) and *tkr1* seedlings grown on medium with 0–10 μM TK VI. Data shown are averages with SD (*n*>20). (B) Phenotyping of 6 DAG seedlings of the WT, *tkr1* mutant, and F_1_ progeny from a cross between the wild type and *tkr1* grown on medium without (MS) or with 3 μM TK VI. (C) Root meristem size of WT and *tkr1* seedlings grown on medium without (Control) or with 3 μM TK VI at 2–9 DAG. Data shown are averages with SD (*n*=20). (D) Root meristems of 9 DAG WT and *tkr1* seedlings grown on medium without (MS) or with 3 μM TK VI. The meristem zone is marked with a vertical black line. The black arrowhead indicates the boundary between the meristem and the elongation zone. The black box is an enlarged image of the boundary between the meristem zone and the elongation zone. (E) Cortical cell length in the differentiation zone of 9 DAG WT and *tkr1* seedlings grown on medium without (Control) or with 3 μM TK VI. Data shown are averages with SD (*n*=20). Asterisks denote Student’s *t*-test significance compared with untreated plants: ***P<0.001. (F) Images of the differentiation zone of the seedlings described in (E). Black arrowheads indicate the length of a single cortical cell. Scale bars=5mm (B), 50 µm (D, F).

To investigate further the mechanisms involved in the TK VI-induced inhibition effect on *Arabidopsis* roots, *Arabidopsis* TK VI-resistant (*tkr*) mutants were screened from EMS-mutagenized M_2_ seedlings. Among a number of putative M_3_ mutants, *tkr1* showed significant TK VI endurance capacity compared with the wild type when treated with 1–5 μM TK VI (especially 3 μM), which could be seen in the primary root length (Fig. 5A, B). The meristem size of the *tkr1* mutant was approximately the same as that of the wild type at 6 DAG (days after germination) when cultivated on half-strength MS medium. However, the meristem zone of the wild type vanished at 4 DAG when 3 μM TK VI was applied, while the meristem cell number of TK VI-treated *tkr1* seedlings only decreased by ~20% compared with untreated *tkr1* seedlings even at 9 DAG (Fig. 5C, D). In *tkr1* seedlings, the length of cortical cells in the differentiation zone was not affected by 3 μM TK VI (Fig. 5E, F). Taken together, we conclude that *tkr1* is a dose-dependent TK VI-resistant mutant.

A genetic analysis revealed that the phenotype of the F_1_ generation from a cross between *tkr1* and the wild type mimics that of *tkr1* (Fig. 5B). F_2_ plants showed a 3:1 segregation ratio of *tkr1* over the wild type (Supplementary Table S2). These results indicate that *tkr1* harbors a monogenic dominant mutation in a nuclear gene.

### Map-based cloning of *GORK*, which encodes a gated outwardly rectifying K^+^ channel protein

**Fig. 6.**
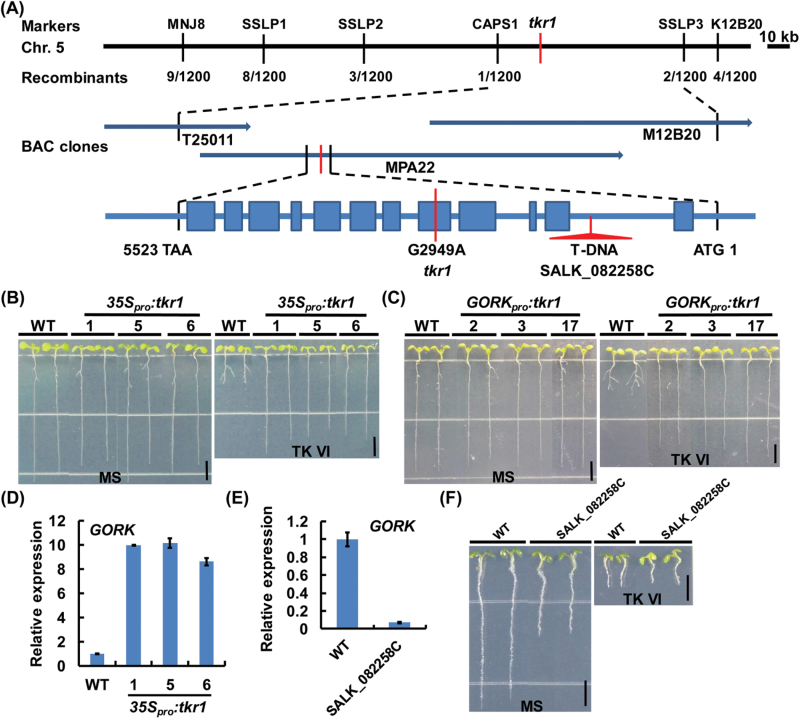
Map-based cloning of the *GORK* gene. (A) Fine genetic and physical mapping of *GORK*. The target gene was initially mapped to a genetic interval between the markers MNJ8 and K12B20 on *Arabidopsis* chromosome 5. The analysis of a mapping population consisting of 1200 plants identified the target gene encoding GORK (At5g37500). In the *GORK* structure illustration, the filled boxes represent exons and the lines represent introns. Mutation and T-DNA insertion sites are shown in red. (B) Wild-type (WT) and *35S*_*pro*_ *:tkr1* seedlings grown on medium without (MS) or with 3 μM TK VI at 6 DAG. (C) WT and *GORK*_*pro*_ *:tkr1* seedlings grown on medium without (MS) or with 3 μM TK VI at 6 DAG. (D) qRT–PCR analysis of *GORK* expression in *35S*_*pro*_ *:tkr1* seedlings. (E) qRT–PCR analysis of *GORK* expression in SALK_082258C. For (D) and (E), 10-day-old seedlings were harvested for RNA extraction and qRT–PCR analysis. The transcript level of *GORK* in the WT was arbitrarily set to 1. The error bars represent the SD of triplicate reactions. (F) WT and SALK_082258C seedlings grown on medium without (MS) or with 3 μM TK VI at 6 DAG. Scale bars=5mm (B, C, F).

Map-based cloning was carried out using an F_2_ population. The mutated gene was determined to be located within bacterial artificial chromosome (BAC) clone MPA22 of chromosome 5 and was ultimately identified as encoding a gated outwardly rectifying K^+^ channel (GORK) protein (Fig. 6A). GORKs can form tetrameric gated outwardly rectifying K^+^ channels, which belong to the shaker family of K^+^ channels. Such channels are activated upon membrane depolarization (Ache *et al.*, 2000; Mäser *et al.*, 2001).

Sequence analysis showed that a single nucleotide substitution in the *tkr1* allele, A^2949^ for G^2949^ (Fig. 6A), results in a change of Gly^313^ to Ser^313^ in the amino acid sequence of GORK. The K^+^ channels of the shaker family possess six transmembrane segments (S1–S6) and a pore-forming domain (P) between S5 and S6 (Jan and Jan, 1992; Gaymard *et al.*, 1998). SKOR is another member of the shaker family of K^+^ channels, and has the closest phylogenetic relationship to GORK based on phylogenetic analysis (Mäser *et al.*, 2001). A protein sequence alignment between GORK and SKOR (Gaymard *et al.*, 1998) indicated that Gly^313^ is next to the last amino acid residue of S6 and links S6 with the C-terminal cytoplasmic region of the subunit (Supplementary Fig. S6). We then constructed *35S*_*pro*_ *:tkr1* and *GORK*_*pro*_ *:tkr1* transgenic lines in the wild-type background and analyzed their response upon TK VI treatment. These lines all showed significantly increased TK VI resistance compared with that of the wild type (Fig. 6B, C), confirming our mapping result. The overexpression of the mutated *GORK* gene in the *35S*_*pro*_ *:tkr1* transgenic lines was verified by qRT–PCR (Fig. 6D).

We tested the TK VI resistance of the T-DNA insertion line SALK_082258C, which contains a T-DNA insertion in the first intron of *GORK* (Fig. 6A). The T-DNA insertion in SALK_082258C disrupts the transcription of *GORK* (Fig. 6E). Unlike *tkr1*, the *GORK* knockout T-DNA insertion line displayed a wild-type response upon TK VI treatment (Fig. 6F). Similarly, the exogenous application of the K^+^ channel blockers TEA^+^ or Ba^2+^ (Demidchik *et al.*, 2010) did not alleviate the inhibition effect of TK VI on primary root growth (Supplementary Fig. S7). We then constructed *35S*_*pro*_ *:GORK* transgenic lines and examined their response to TK VI treatment. All of the *GORK* overexpression lines showed a wild-type response when treated with TK VI (Supplementary Fig. S8). Together, these observations indicate that the TK VI-enduring ability of *tkr1* is not the result of either the loss of function of *GORK* or its overexpression, indicating that the single amino acid substitution of Ser^313^ for Gly^313^ most probably changes the function of GORK channels.

### TK VI represses the outward K^+^ currents mediated by GORK channels, resulting in the disruption of auxin gradients

**Fig. 7.**
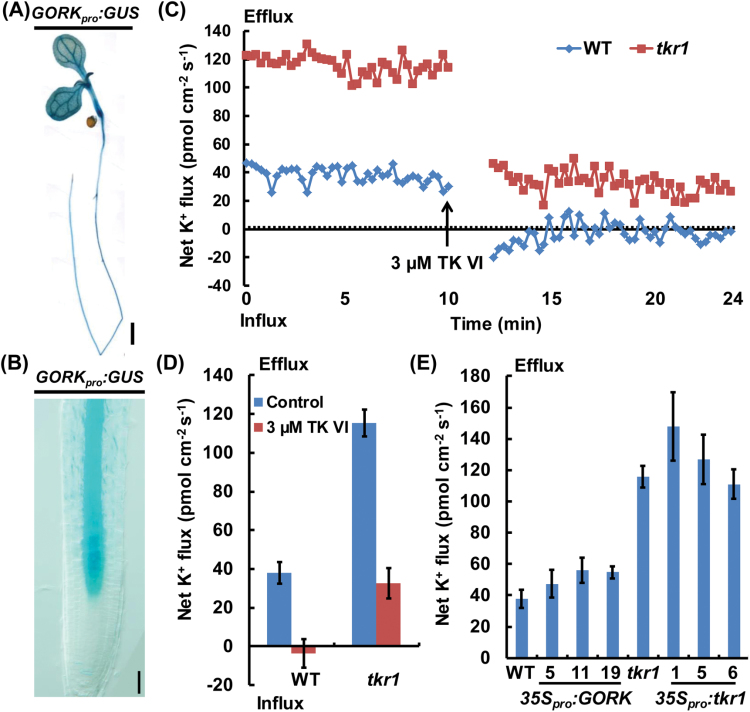
K^+^ flux analysis using NMT. (A, B) *GORK*_*pro*_ *:GUS* expression pattern in seedlings at 5 DAG. (C) Transient K^+^ flux measurements upon TK VI (3 μM) shock in the elongation zone of the wild type (WT) and *tkr1.* Before the TK VI shock, steady K^+^ flux was examined for 10min. The data in the following 2min after TK VI shock are not shown for TK VI diffusion. Data shown are representative of at least three independent experiments. Each point represents the mean of six seedlings. (D) Mean efflux of K^+^ before and after TK VI shock in the WT and *tkr1* seedlings described in (C). Data shown are averages with the SD (*n*=6) and are representative of at least three independent experiments. (E) Mean efflux of K^+^ in WT, *tkr1*, *35S*_*pro*_ *:GORK*, and *35S*_*pro*_ *:tkr1* seedlings under control conditions. Steady K^+^ efflux in the indicated seedlings was examined for 15min. Data shown are averages with the SD (*n*=6) and are representative of at least three independent experiments. Scale bar=5mm (A), 50 µm (B).

The function of K^+^ efflux through GORK channels in stomatal closure has been studied in detail (Hosy *et al.*, 2003). Recent data have demonstrated that GORK-mediated K^+^ efflux is tightly linked to the production of stress-induced reactive oxygen species and may also be involved in programmed cell death (Demidchik *et al.*, 2010). We first investigated the effect of the mutation in *tkr1* on K^+^ flux before and after TK VI treatment using NMT. As indicated by our *GORK*_*pro*_ *:GUS* reporter, *GORK* was expressed throughout the entire root except the root tip (Fig. 7A, B), in agreement with previous reports (Ache *et al.*, 2000; Ivashikina *et al.*, 2001). Because GORK-mediated K^+^ efflux is more sensitive to outer stimuli in the elongation zone than in the mature area (Demidchik *et al*; 2010), all of the measurements were carried out in the elongation zone of the primary root. Without TK VI treatment, an average K^+^ efflux of ~38 pmol cm^−2^ s^−1^ was recorded in the wild type, while the K^+^ efflux in *tkr1* was three times higher than this value under the same conditions. Upon TK VI shock, the K^+^ efflux in the wild type was almost completely turned off, and an average K^+^ efflux of ~33 pmol cm^−2^ s^−1^ still remained in *tkr1* despite a severe reduction (Fig. 7C, D). K^+^ flux was also measured in several independent *35S*_*pro*_ *:GORK* and *35S*_*pro*_ *:tkr1* transgenic lines under control conditions. The K^+^ efflux rate in *35S*_*pro*_ *:GORK* lines was similar to that of the wild-type seedlings, and the K^+^ efflux rate in *35S*_*pro*_ *:tkr1* lines resembled that of the *tkr1* seedlings (Fig. 7E). These results indicate that the overexpression of *GORK* in *35S*_*pro*_ *:GORK* transgenic lines has no obvious effect on K^+^ efflux modulation, which is in agreement with their wild-type response upon TK VI treatment.

**Fig. 8.**
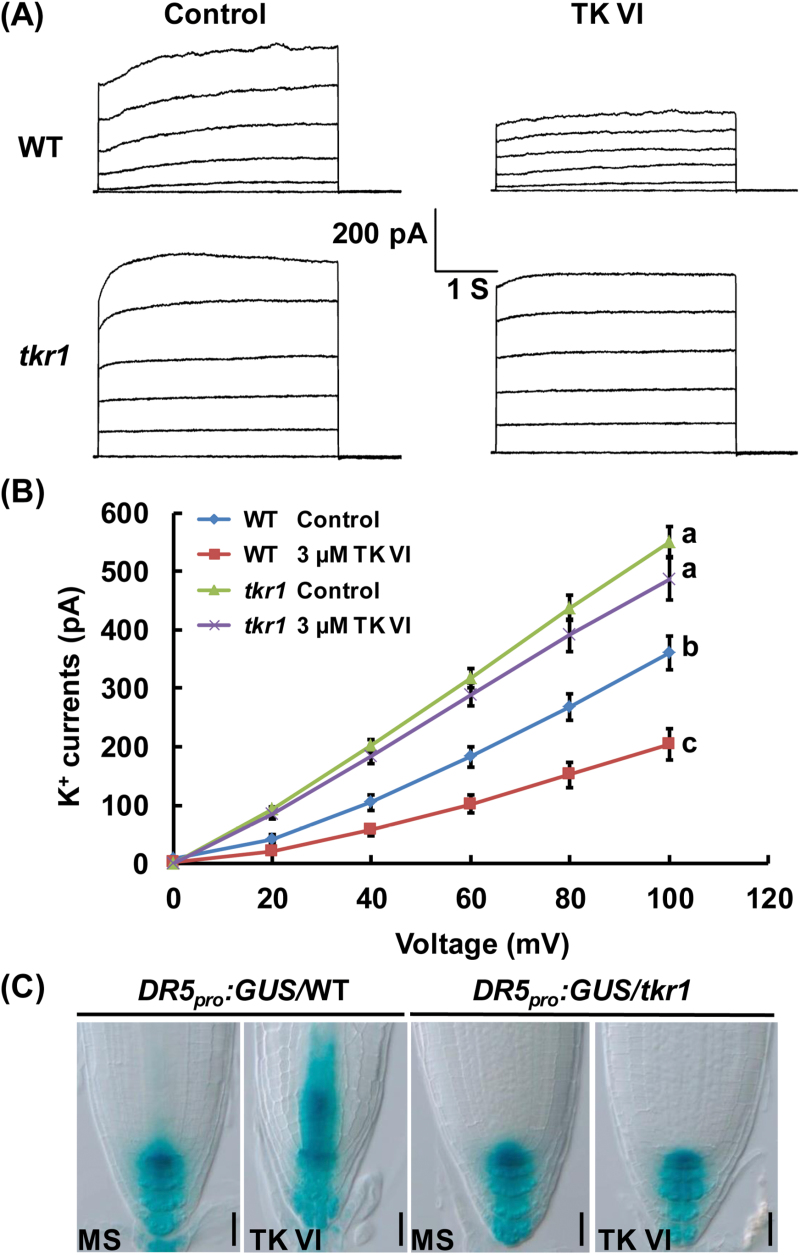
The effect of TK VI on outward K^+^ currents as analyzed by patch-clamp whole-cell recordings. (A) Patch-clamp whole-cell recordings of outward K^+^ current traces in wild-type (WT) and *tkr1* root cell protoplasts without (Control) or with 3 μM TK VI. Time and current scales shown in the middle apply to all traces. (B) The current/voltage relationship of the whole-cell outward K^+^ currents as illustrated in (A). The number of protoplasts measured for the curves were 16 (for WT), 14 (for WT treated with 3 μM TK VI), 11 (for *tkr1*), and 11 (for *tkr1* treated with 3 μM TK VI). Data shown are averages with the SD. Samples with different letters are significantly different; *P*<0.001. (C) Effect of TK VI on auxin distribution gradients in the WT and *tkr1*. *DR5*_*pro*_ *:GUS*/WT and *DR5*_*pro*_ *:GUS/tkr1* seeds were germinated on medium without (MS) or with 3 μM TK VI for 5 DAG before GUS staining assays were performed. Scale bars=50 µm.

To confirm further the TK VI-induced suppression of K^+^ efflux, we conducted patch-clamp whole-cell recordings using root cell protoplasts isolated from the primary root elongation zone epidermis. In the absence of TK VI, outward K^+^ currents in *tkr1* were much larger than those in the wild type; in the presence of 3 µM TK VI, outward K^+^ currents were significantly reduced in the wild type and only slightly suppressed in *tkr1* (Fig. 8A, B).

Taken together, these results indicate that TK VI negatively regulates GORK-mediated K^+^ efflux. The substitution of Ser^313^ for Gly^313^ in the GORK amino acid sequence results in an enhanced K^+^ efflux magnitude and alleviates TK VI-induced suppression of K^+^ efflux in roots.

To test whether the TK VI-induced disruption of auxin response gradients in the *Arabidopsis* root tip is related to the suppression of GORK-mediated K^+^ efflux, we introduced the *DR5*_*pro*_ *:GUS* reporter into the *tkr1* background and analyzed the effect of TK VI on auxin distribution in the root tip. As shown by the *DR5*_*pro*_ *:GUS* reporter, without TK VI treatment, the *DR5*_*pro*_ *:GUS* expression pattern in the root tip of the *tkr1* seedlings was the same as that in the root tip of the wild-type seedlings. After growing on medium supplied with 3 µM TK VI for 5 DAG, the auxin response gradients in the wild-type seedlings were badly disrupted, with the auxin maximum lying above the QC/columella initial region, while auxin distribution in the root tip of the TK VI-treated *tkr1* seedlings remained the same as that in the untreated *tkr1* seedlings (Fig. 8C). These results indicate that the TK VI-induced disruption of auxin gradients in the *Arabidopsis* root tip results from TK VI-induced suppression of GORK-mediated K^+^ efflux.

### The *tkr1* mutant is also resistant to alamethicin, the most studied long-sequence peptaibol

**Fig. 9.**
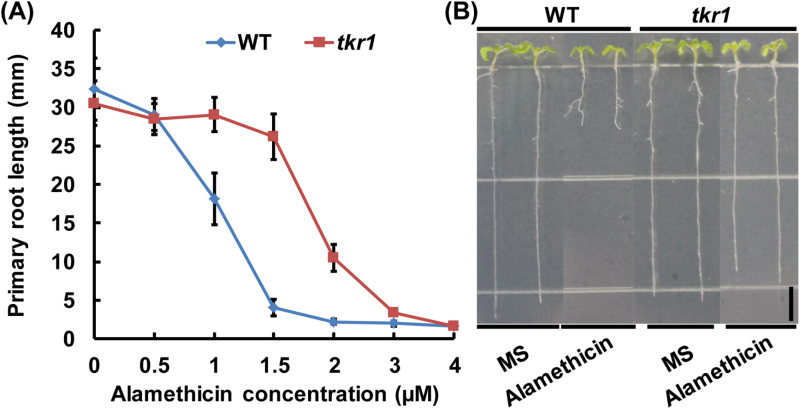
Alamethicin effects on primary root growth in the wild type (WT) and *tkr1* mutant. (A) Primary root length of 6 DAG WT and *tkr1* seedlings grown on medium with 0–4 μM alamethicin (Sigma, purity ≥98%). Data shown are averages with the SD (*n*>20). (B) WT and *tkr1* seedlings grown on medium without (MS) or with 1.5 μM alamethicin at 6 DAG. Scale bars=5mm.

Long-sequence peptaibols are thought to function through the formation of ion channels in lipid membranes according to the ‘barrel-stave model’ (Chugh and Wallace, 2001). To determine whether long-sequence peptaibols inhibit plant growth through the same mechanism as TK VI, we investigated the effect of alamethicin on the primary root growth of the wild type and the *tkr1* mutant. We found that alamethicin inhibited the primary root growth of *Arabidopsis* in a dose-dependent manner as previously described (Matic *et al.*, 2005; Rippa *et al.*, 2007, 2010), and the *tkr1* mutant exhibited significantly increased resistance to alamethicin doses of 1–3 µM (especially 1.5 µM) (Fig. 9A, B). This result indicates that alamethicin probably inhibits plant growth through the same mechanism as TK VI.

## Discussion

### TK VI-induced inhibition of primary root growth is a complex process involving multiple cellular and molecular processes

Our results indicated that TK VI inhibited both cell division and cell elongation in *Arabidopsis* roots; the maintenance of the root stem cell niche was also disrupted by TK VI. According to our data, TK VI-induced auxin accumulation (at 3h) occurs much earlier than the TK VI-induced disruption of auxin response gradients (at 24h) in *Arabidopsis* root tips, which means that the early accumulation of auxin in the root tip does not affect auxin distribution gradients. The disruption of auxin response gradients is probably caused by the inhibition of polar auxin transport in the root tip, which results in the reduction of auxin being transported to the QC/columella initial region to maintain the auxin maximum. TK VI severely affected the auxin content and auxin response gradients in wild-type *Arabidopsis* root tips. However, unlike *axr1-1*, which is an auxin-resistant mutant (Lincoln *et al.*, 1990), *tkr1* is not resistant to IAA (Supplementary Fig. S9A). In addition, in the tested auxin-related mutants, including the auxin signaling pathway mutant *axr1-1*, the auxin transport mutants *pin1-5*, *pin2 pin3 pin4*, *pin2 pin3 pin7*, and *pin3 pin4 pin7*, and the auxin biosynthesis mutant *asa1-1*, no TK VI resistance was observed (Supplementary Fig. S9B). These results indicate that auxin-related pathways are not the direct or specific targets of TK VI in the *Arabidopsis*–TK VI interaction process, and other key regulators of root patterning are also involved. PLETHORA (PLT) proteins are auxin-induced transcription factors that are essential for root stem cell niche patterning and root meristem activity (Aida *et al.*, 2004; Mähönen *et al.*, 2014). The expression of *PLT1* and *PLT2* was not notably affected during the first 12h of TK VI treatment, but was dramatically decreased after 24h of treatment (Supplementary Fig. S10A–E). The expression of *SHORT-ROOT* (*SHR*) and *SCARECROW* (*SCR*), which act in parallel with the PLT pathway to provide positional information for the stem cell niche (Helariutta *et al.*, 2000; Sabatini *et al.*, 2003), was also remarkably suppressed (Supplementary Fig. S10F, G). These data suggest that TK VI-induced inhibition of root growth is a complex process that involves multiple cellular and molecular processes.

### K^+^ homeostasis is important for the maintenance of auxin gradients

As one of the most abundant and irreplaceable macronutrient in plants, K^+^ plays crucial roles in many fundamental processes in living cells, such as electrical neutralization, osmoregulation, membrane potential regulation, and the activation of crucial enzymatic reactions (Clarkson and Hanson, 1980). TRH1, a member of the AtKT/AtKUP/HAK K^+^ transporter family, is required for polar auxin transport in *Arabidopsis* roots (Rigas *et al.*, 2001; Vicente-Agullo *et al.*, 2004). Although the physiological mechanism by which TRH1 interacts with root auxin transport remains to be clarified, a connection exists between K^+^ transport and polar auxin transport. As demonstrated using the *DR5*_*pro*_ *:GUS* reporter, TK VI-induced disruption of auxin gradients was recovered to normal levels in the *tkr1* mutant, in which the function of GORK channels is altered due to a substitution of Ser^313^ for Gly^313^. Our data hint at an inter-relationship between GORK channels and the maintenance of auxin homeostasis, though the mechanism involved is unclear. The *trh1* mutant phenotype was not restored by high external K^+^ concentrations (Rigas *et al.*, 2001); similarly, TK VI-induced inhibition of root growth was not rescued by 50mM external K^+^ (Supplementary Fig. S11). Additionally, trilongins, peptaibols also from *T. longibrachiatum*, are reported to form voltage-dependent Na^+^/K^+^-permeable channels to cause mammalian cell toxicity (Mikkola *et al.*, 2012). These data suggest that TK VI-induced disruption of auxin gradients is not due to K^+^ starvation; instead, this disruption is most probably caused by alterations in K^+^ homeostasis.

We applied both NMT and patch-clamp whole-cell recordings to analyze the effect of the substitution of Ser^313^ for Gly^313^ on the function of GORK channels. According to these data, K^+^ efflux is suppressed by TK VI in the wild type, and this TK VI-induced suppression is much alleviated in *tkr1*, although how the mutation changes the function of GORK channels is unclear. It is well known that some K^+^ channels in animal cells are regulated by specific kinases (Jan and Jan, 1997). The function of AKT1, another member of the shaker family of K^+^ channels (Mäser *et al.*, 2001), has been reported to be directly regulated by protein phosphorylation (Xu *et al.*, 2006). It is unknown whether GORK is regulated by phosphorylation and whether the change of Gly^313^ to Ser^313^, an amino acid that can be phosphorylated by protein kinase, affects the phosphorylation of GORK, which merits further study.

### Mechanisms of peptaibol-induced inhibition of primary root growth in Arabidopsis

Based on the results of this study and those of other studies, the mechanisms of TK VI-induced inhibition of primary root growth in *Arabidopsis* can be proposed. When *Arabidopsis* seedlings are treated with TK VI, ion channels are formed in the plasma membrane of the root cells and the ion balance of root cells is disrupted. GORK channels somehow sense this disruption in ion balance and act immediately to decrease K^+^ efflux. This alteration in K^+^ homeostasis reduces meristem size by suppressing cell division, enhances auxin content by activating local auxin biosynthesis, and disrupts auxin gradients by suppressing polar auxin transport in the root tip. The TK VI-induced disruption of auxin homeostasis and other key regulators involved in root patterning, such as *PLT1/PLT2* and *SHR*/*SCR*, results in the disruption of maintenance of the root stem cell niche. Post-embryonic root growth is ultimately terminated by TK VI due to a lack of a root stem cell niche, which serves as the source of all differentiated cell types during root development. In addition, the TK VI-resistant mutant *tkr1* is also resistant to alamethicin, which suggests that long-sequence peptaibols probably inhibit primary root growth in *Arabidopsis* via the same mechanism as TK VI.

## Supplementary data

Supplementary data are available at *JXB* online.

Figure S1. Construction of the Δ*Tpx1* mutant by knocking out the *Tpx1* gene that encodes the NRPS responsible for TKs biosynthesis in SMF2.

Figure S2. *Arabidopsis* (Col-0) seedlings grown in soil supplied with different concentrations of spores from the wild-type SMF2 or the Δ*Tpx1* mutant.

Figure S3. Biomass loss in TK VI-treated *Arabidopsis* (Col-0) shoots and roots.

Figure S4. TK VI-induced expression of *ASA1* and *ASB1* analyzed by promoter–GUS reporters.

Figure S5. TK VI effect on the expression of the auxin efflux transporter genes.

Figure S6. Location of GORK Gly^313^ by amino acid sequence alignment with SKOR.

Figure S7. Effect of K^+^ channel blockers on TK VI-induced inhibition of primary root growth in wild-type *Arabidopsis* (Col-0) and *tkr1* seedlings.

Figure S8. Phenotyping of independent *35S*_*pro*_ *:GORK* transgenic lines upon TK VI treatment.

Figure S9. The auxin resistance of *tkr1* and phenotyping of auxin-related *Arabidopsis* mutants upon TK VI treatment.

Figure S10. TK VI-induced repression of the expression of *PLT1/PLT2* and *SHR/SCR*.

Figure S11. The effect of 50mM external K^+^ on TK VI-induced inhibition of root growth.

Table S1. DNA primers used for qRT–PCR assays.

Table S2. Genetic analysis of the *tkr1* mutant.

Supplementary Data
