# Supplementary material for: Cellular and molecular insight into the inhibition of primary root growth of Arabidopsis induced by peptaibols, a class of linear peptide antibiotics mainly produced by Trichoderma spp
Source: J Exp Bot. 2016 Feb 5;67(8):2191–205. doi: 10.1093/jxb/erw023 (PMC4809282; doi:10.1093/jxb/erw023)
Supplement: Supplementary Data [file supp_67_8_2191__index.html]

Cellular and molecular insight into the inhibition of primary root growth of Arabidopsis induced by peptaibols, a class of linear peptide antibiotics mainly produced by Trichoderma spp. — Cellular and molecular insight into the inhibition of primary root growth of Arabidopsis induced by peptaibols, a class of linear peptide antibiotics mainly produced by Trichoderma spp. — Supplementary Data 

# Cellular and molecular insight into the inhibition of primary root growth of Arabidopsis induced by peptaibols, a class of linear peptide antibiotics mainly produced by *Trichoderma* spp.

## Supplementary Data

Data files

- supplementary\_figures\_S1\_S11\_table\_S1\_S2.pdf - Supplementary Data
