# Supplementary material for: Cellular and molecular insight into the inhibition of primary root growth of Arabidopsis induced by peptaibols, a class of linear peptide antibiotics mainly produced by Trichoderma spp
Source: J Exp Bot. 2016 Feb 5;67(8):2191–205. doi: 10.1093/jxb/erw023 (PMC4809282; doi:10.1093/jxb/erw023)
Supplement: Supplementary Data [file supp_erw023_supplementary_figures_S1_S11_table_S1_S2.pdf]

Supplementary data

Cellular and Mocecular Insight into the Inhibition of Primary Root Growth of *Arabidopsis* Induced by Peptaibols, a Class of Linear Peptide Antibiotics Mainly Produced by *Trichoderma* spp.

Wei-Ling Shi[1,2], Xiu-Lan Chen[1], Li-Xia Wang[1], Zhi-Ting Gong[1], Shuyu Li[2], Chun-Long Li[3], Bin-Bin Xie[1], Wei Zhang[3], Mei Shi[1], Chuanyou Li[2], Yu-Zhong Zhang[1] and Xiao-Yan Song[1,*]

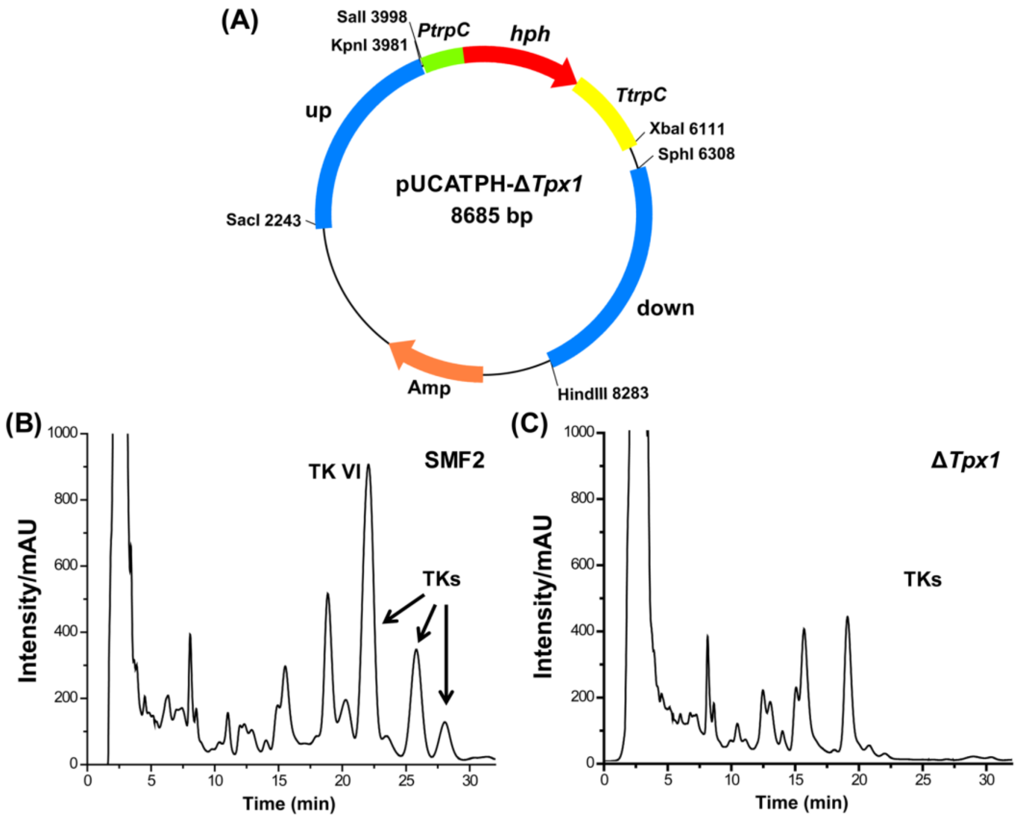

Fig. S1. Construction of the Δ*Tpx1* mutant by knocking out the *Tpx1* gene that encodes the NRPS responsible for TKs biosynthesis in SMF2.

(A) Vector constructed for *Tpx1* knockout. To obtain the mutant with inactivated *Tpx1*, a 1021 bp region of *Tpx1* was replaced by the hygromycin B phosphotransferase (*hph*) gene. To this end, 1739 bp and 1980 bp of the upstream and downstream regions of the *Tpx1* deletion region were amplified and inserted into the pUCATPH vector, respectively. The constructed vector pUCATPH-Δ*Tpx1* was transformed into the SMF2 protoplasts to generate the Δ*Tpx1* strain.

(B) TKs production in wild-type SMF2 analyzed by HPLC.

(C) TKs production in Δ*Tpx1* analyzed by HPLC. The TKs are not detectable in Δ*Tpx1* by HPLC.

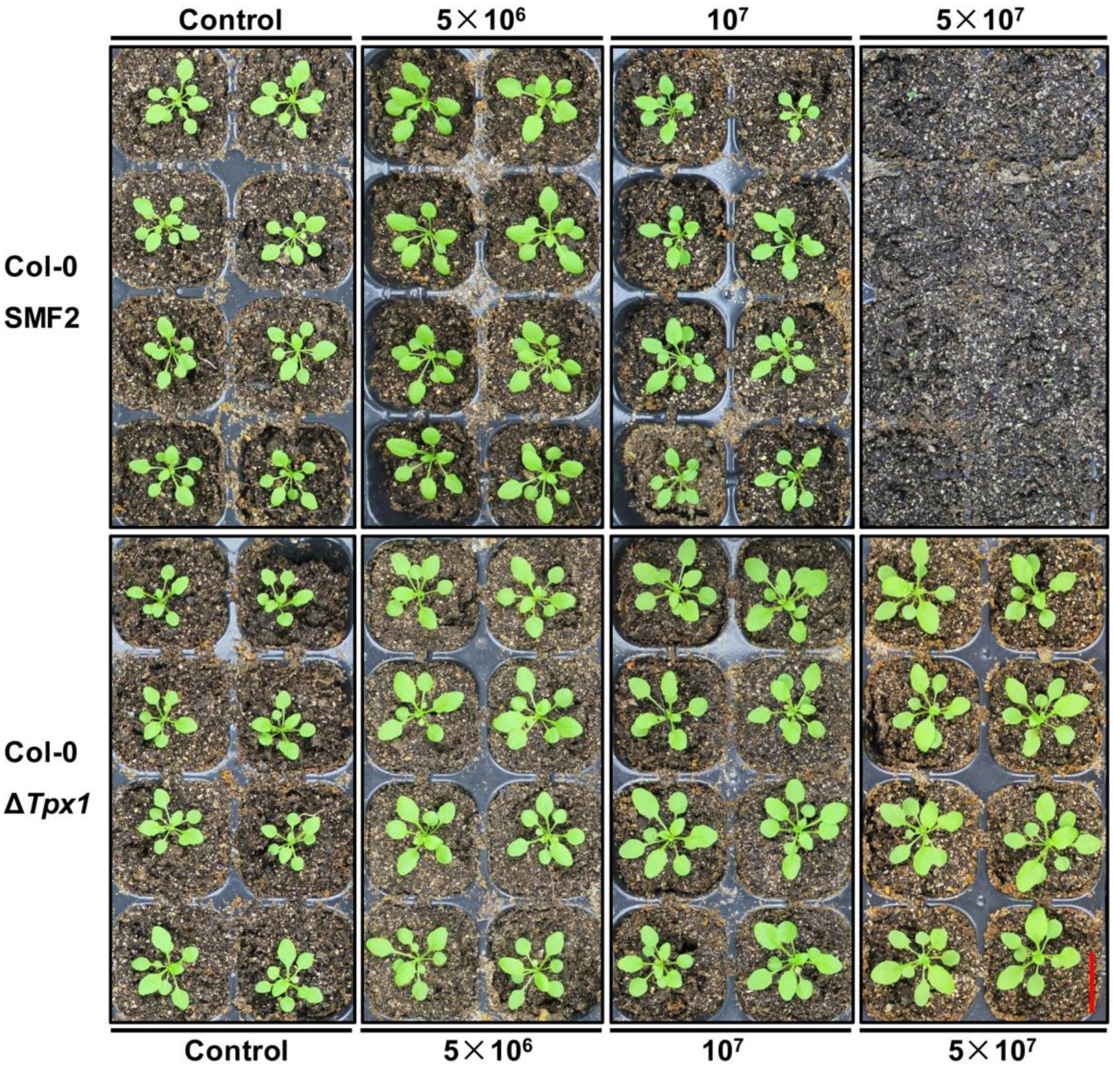

Fig. S2. *Arabidopsis* (Col-0) seedlings grown in soil supplied with different concentrations of spores from the wild-type SMF2 or the Δ*Tpx1* mutant. Eight-day-old Col-0 seedlings were transplanted to soil without (Control) or with the indicated concentrations of spores from wild-type SMF2 and Δ*Tpx1*, respectively, and grown for an additional 2 weeks. Bar = 2 cm.

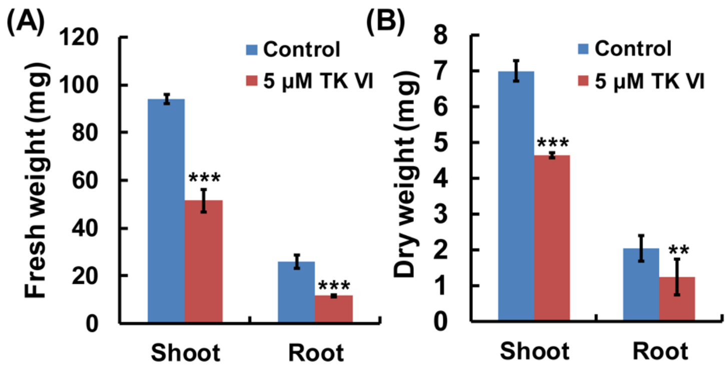

Fig. S3. Biomass loss in TK VI-treated *Arabidopsis* (Col-0) shoots and roots.

(A) Fresh weight of the shoots and roots of 5-DAG Col-0 seedlings grown on medium without (Control) or with 5 μM TK VI.

(B) Dry weight of the shoots and roots of 5-DAG Col-0 seedlings grown on medium without (Control) or with 5 μM TK VI.

For (A, B), Col-0 seeds were grown on medium without or with 5 μM TK VI for 5 DAG before their shoots and roots were cut apart and weighed immediately (A) or weighed after drying at 65°C for 3 d (B). 30 seedlings were weighted at a time and the error bars represent the SD of triplicate measurements. The asterisks denote Student's *t* test significance compared to untreated plants: $**P < 0.01$ and $***P < 0.001$.

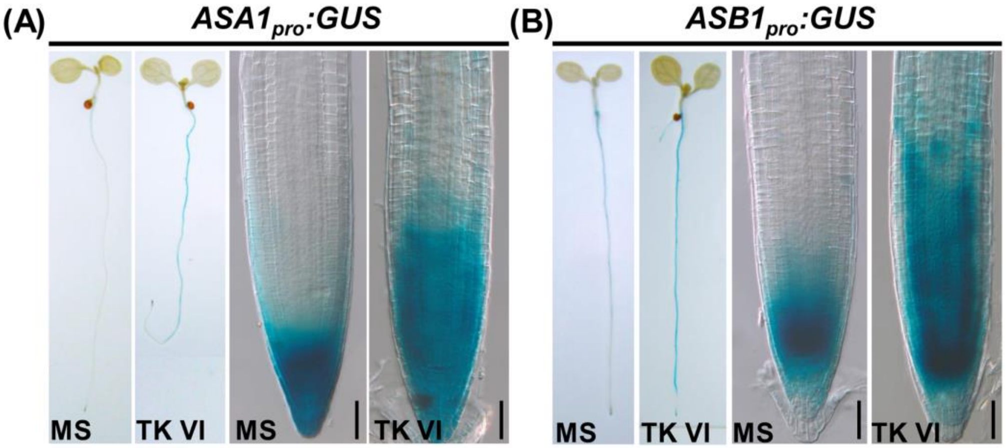

Fig. S4. TK VI-induced expression of *ASA1* and *ASB1* analyzed by promoter-GUS reporters. Six-day-old $ASA1_{pro}$*:GUS* (A) and $ASB1_{pro}$*:GUS* (B) seedlings were transferred to medium without (MS) or with 5 μM TK VI for 12 h ($ASA1_{pro}$*:GUS*) or 3 h ($ASB1_{pro}$*:GUS*) before the GUS staining assays. Bars = 50 μm (A, B).

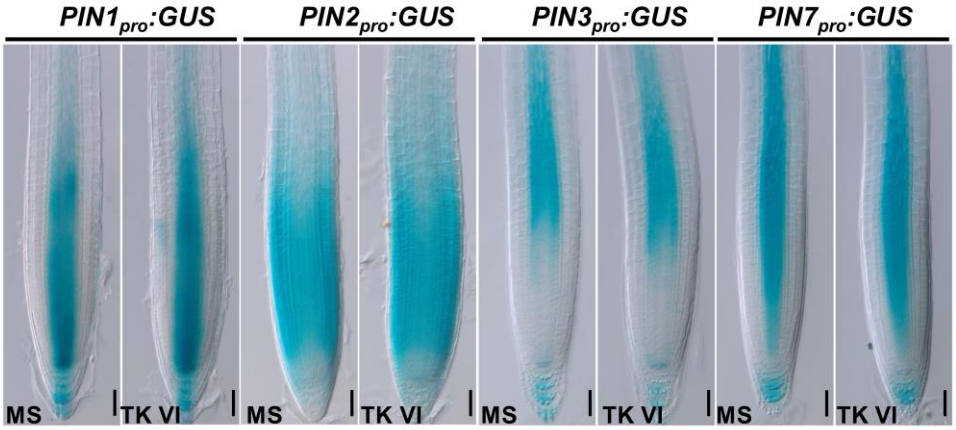

Fig. S5. TK VI effect on the expression of the auxin efflux transporter genes. Six-day-old $PIN1_{pro}$*:GUS*, $PIN2_{pro}$*:GUS*, $PIN3_{pro}$*:GUS* and $PIN7_{pro}$*:GUS* seedlings were transferred to medium without (MS) or with 5 μM TK VI for 3 h before the GUS staining assays. Bars = 50 μm.

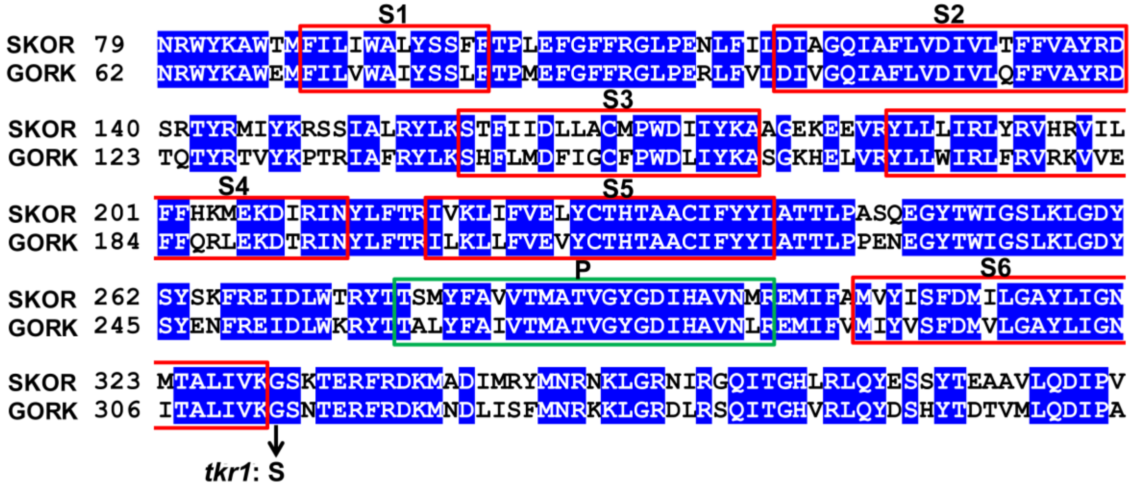

Fig. S6. Location of GORK Gly^{313} by amino acid sequence alignment with SKOR. Sequence alignment was performed with Clustal X program. Amino acid residues surrounded by blue are identical between SKOR and GORK. S1-S6 are indicated in red boxes. The green box represents the P domain.

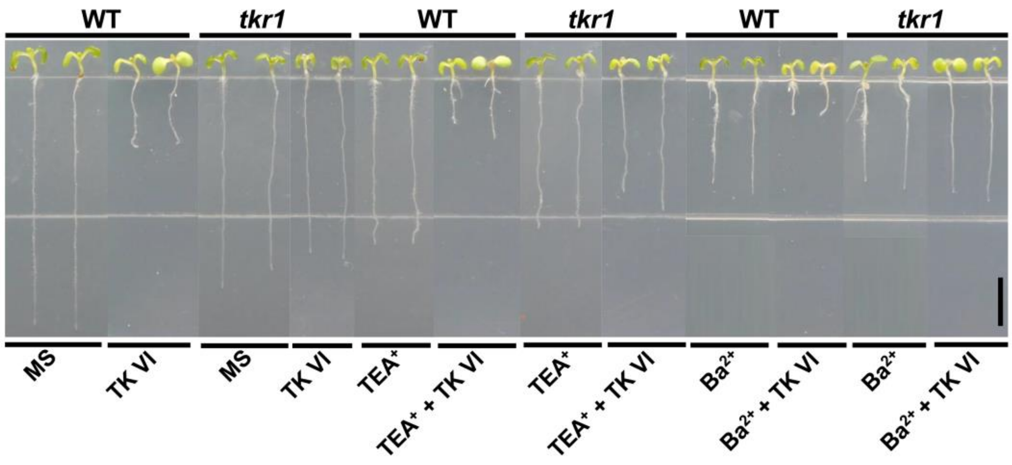

Fig. S7. Effect of K^+ channel blockers on TK VI-induced inhibition of primary root growth in the wild type *Arabidopsis* (Col-0) and *tkr1* seedlings. The wild-type (WT) and *tkr1* seeds were germinated on medium without or with 3 µM TK VI in the presence of 4 mM TEA^+ or 4 mM Ba^{2+} for 6 DAG. Bar = 5 mm.

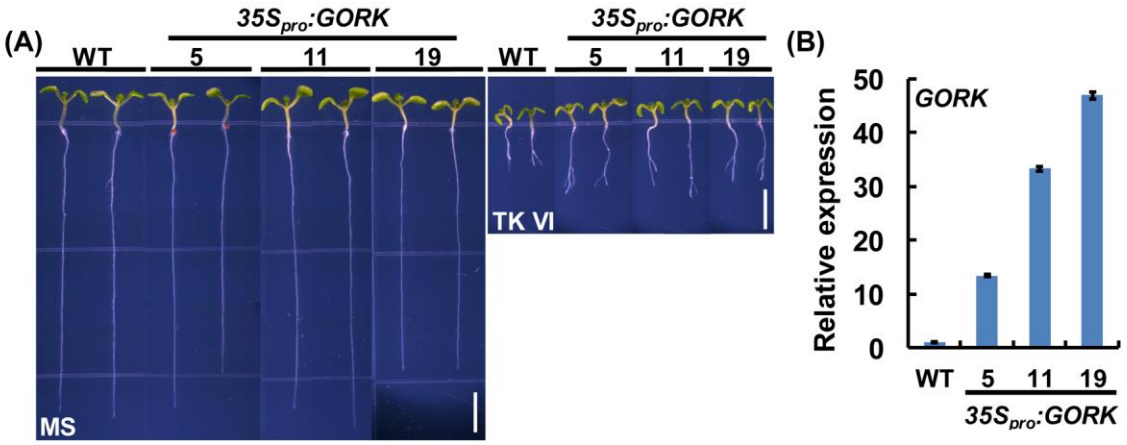

Fig. S8. Phenotyping of independent *35S$_{pro}$:GORK* transgenic lines upon TK VI treatment.

(A) Wild type (WT) and several independent *35S$_{pro}$:GORK* transgenic lines grown on medium without (MS) or with 3μM TK VI at 7 DAG. Bars = 5 mm.

(B) qRT-PCR analysis of *GORK* expression in the indicated seedlings. Ten-day-old seedlings were harvested for RNA extraction and qRT-PCR analysis. The transcript level of *GORK* in the wild type (WT) was arbitrarily set to 1. The error bars represent the SD of triplicate reactions.

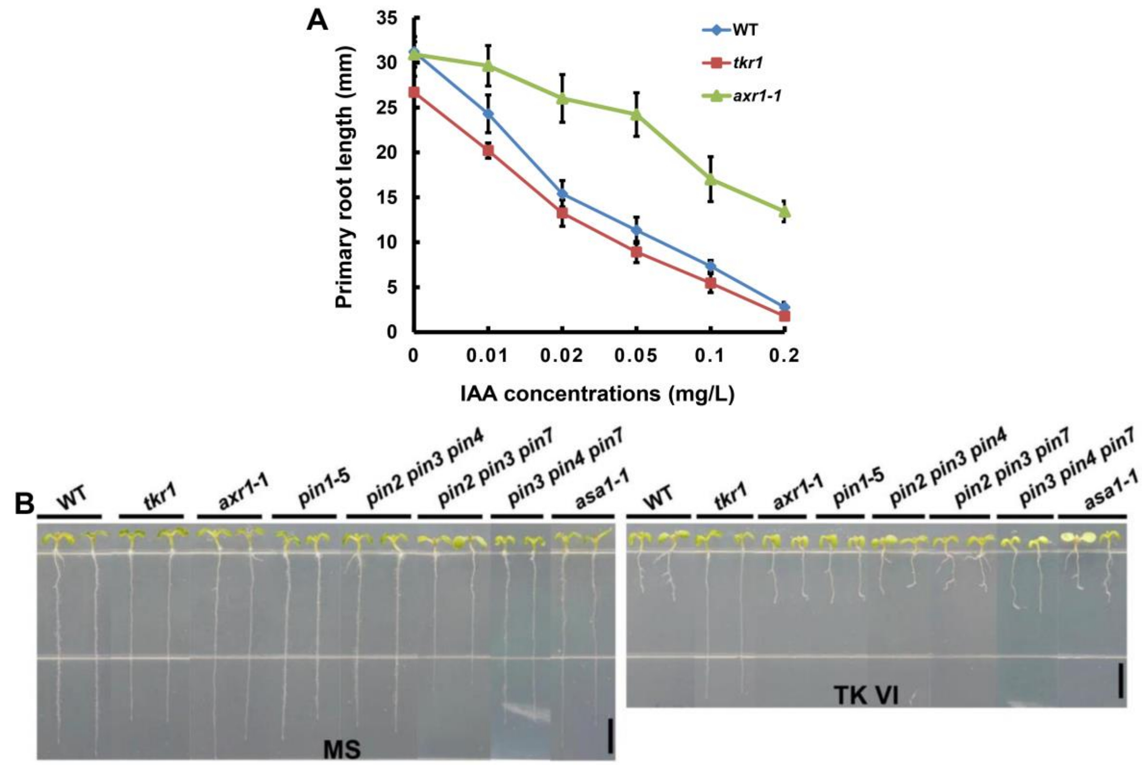

Fig. S9. The auxin resistant ability of *tkr1* and phenotyping of auxin-related *Arabidopsis* mutants upon TK VI treatment.

(A) Primary root length of 5-DAG wild type (WT), *tkr1* and *axr1-1* seedlings grown on medium with 0-0.2 mg/L IAA (Sigma, purity ≥ 98%). Data shown are averages with SD (*n* > 20).

(B) Phenotyping of auxin-related *Arabidopsis* mutants upon TK VI treatment. The indicated seedlings were grown on medium without (MS) or with 3 μM TK VI for 6 DAG. Bars = 5 mm.

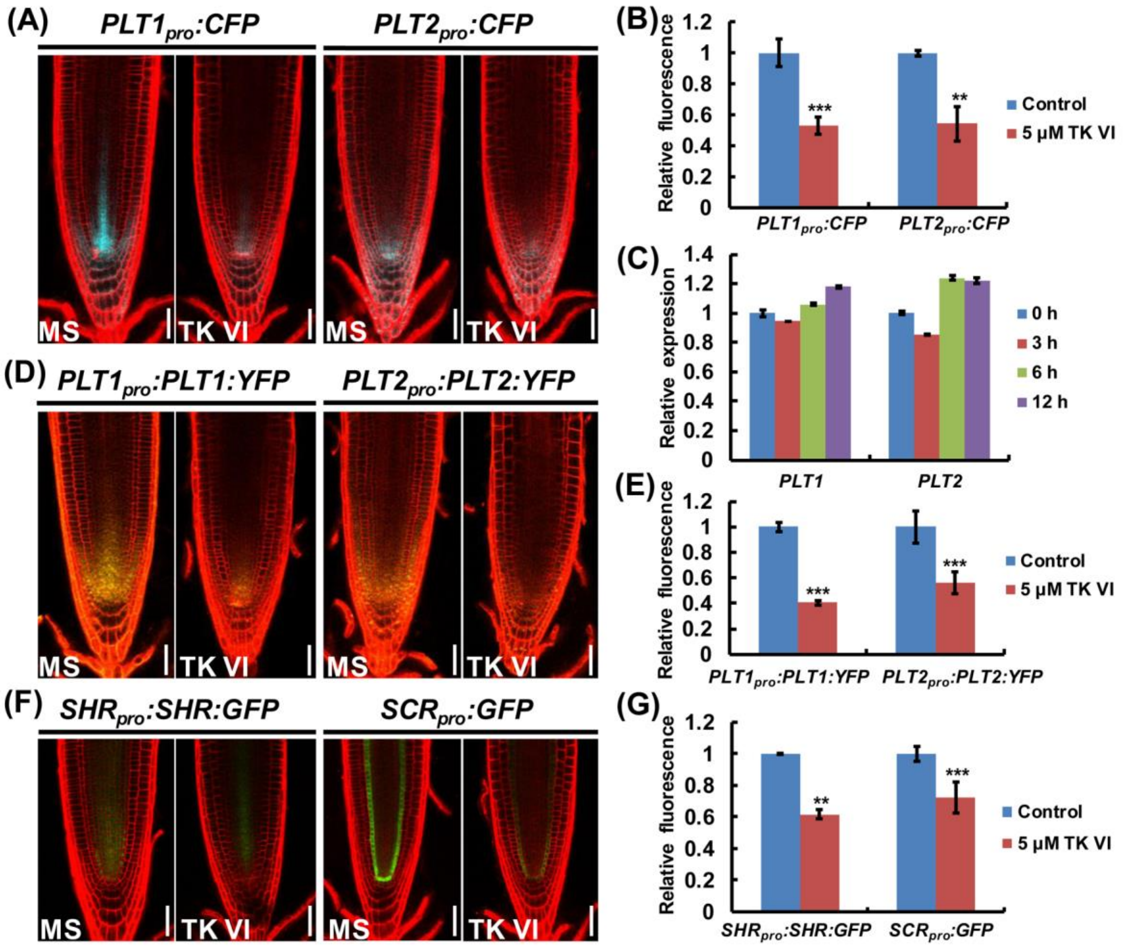

Fig. S10. TK VI-induced repression of the expression of *PLT1/PLT2* and *SHR/SCR*.

(A) TK VI reduces the expression of *PLT1*$_{pro}$*:CFP* and *PLT2* $_{pro}$*:CFP* in Col-0.

(B) Quantification of the CFP fluorescence shown in (A).

(C) Time-course expression of *PLT1* and *PLT2* in response to TK VI treatment. Six-day-old Col-0 seedlings were transplanted to medium without or with 5 μM TK VI for the indicated time periods, and the 2-mm root tips were harvested for RNA extraction and qRT-PCR analysis. Transcript levels of *PLT1* and *PLT2* were normalized to *ACTIN2* expression. The transcript levels of *PLT1* and *PLT2* without TK VI treatment were arbitrarily set to 1. Results of one of three independent experiments are shown. Error bars represent the SD of triplicate reactions.

(D) TK VI-induced repression of the expression of *PLT1$_{pro}$:PLT1:YFP* and *PLT2$_{pro}$:PLT2:YFP* in Col-0.

(E) Quantification of the YFP fluorescence shown in (D).

(F) TK VI-induced repression of the expression of *SHR$_{pro}$:SHR:GFP* and *SCR$_{pro}$:GFP* in Col-0.

(G) Quantification of the GFP fluorescence shown in (F).

For (A, B, D, E, F, G), six-day-old seedlings were transplanted to medium without (MS/Control) or with 5 μM TK for 24 h before the CFP/YFP/GFP fluorescence was monitored. Data shown are averages with SD (n = 15). The asterisks denote Student's *t* test significance compared to untreated plants: **P < 0.01 and ***P < 0.001. Bars = 50μm (A, D, F).

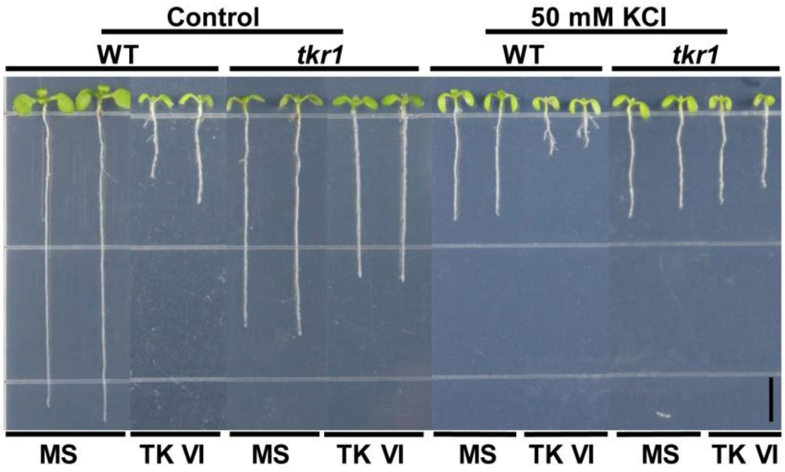

Fig. S11. The effect of 50 mM external K^+ on TK VI-induced inhibition of root growth. Wild type (WT) and *tkr1* seedlings were grown on the indicated medium for 7 DAG.

Table S1. DNA primers used for qRT-PCR assays.

Primer name	Sequence
ACT2-Forward:	5'-TTGACTACGAGCAGGAGATGG-3'
ACT2-Reverse:	5'-ACAAACGAGGGCTGGAACAAG-3'
CYCB1;1-Forward:	5'-CCGGAACTGAATCTGCTTAGG-3'
CYCB1;1-Reverse:	5'-GCGACTCATTAGACTTGTTCA-3'
CYCB1;4-Forward:	5'-ACGTGGAATCGCAGGTGAAATC-3'
CYCB1;4-Reverse:	5'-AGCCTTGCTTCGAGCTCTTAAG-3'
CYCA1;1-Forward:	5'-TCACTAGTAGCTGCTTCCGCCATT-3'
CYCA1;1-Reverse:	5'-ACACATCCTCTCAACTCCATCGCT-3'
CYCD3;1-Forward:	5'-CATCGTTGAACAGTCCAAGCTGC-3'
CYCD3;1-Reverse:	5'-TACGATTGCCCATGGCAGATGC-3'
E2FA-Forward:	5'-AATGGGCGAAATAGCACCAACAGC-3'
E2FA-Reverse:	5'-TTGTATGGAACGCACCTGCCATTG-3'
MCM3-Forward:	5'-AACACCAAGTGGACGTAGAGGCAA-3'
MCM3-Reverse:	5'-TTCAATCCTTGCTGCAGAGACCGT-3'
PCNA1-Forward:	5'-TGGGTTACATTCGTTACTAC-3'
PCNA1-Reverse:	5'-ATACAAAGGAATCTCACCA-3'
CDKB1;1-Forward:	5'-AGATGGTTCGGAGGCAAGCTCTTT-3'
CDKB1:1-Reverse:	5'-TAGGGTAAACATGCCAGTCACGCA-3'
KRP1-Forward:	5'-GAGAAGGCGAAATTGATGACG-3'
KRP1-Reverse:	5'-TCTAATGGCTTCTCCTTCTCG-3'
KRP2-Forward:	5'-AGAGATCTGGAAGGTGACGTCGTA-3'
KRP2-Reverse:	5'-AATTTCTCGCCACAATTCCACCGC-3'
E2FC-Forward:	5'-TCCCACGGTTTCAGAACCAGACAT-3'
E2FC-Reverse:	5'-CAACTTGTCGCTTGTTTCCGCACT-3'
IAA2-Forward:	5'-AGTCAACGAGCTTAACCTTAAG-3'
IAA2-Reverse:	5'-TCACGAGTTTCCTCAAATAGAC-3'
ASA1-Forward:	5'-GTAGAGAAGCTTATGAACATCGA-3'
ASA1-Reverse:	5'-GGTGCACCACTAACTGTTCCCAC-3'
ASB1-Forward:	5'-GGGGAAGAGTCGTAGAGATGTCT-3'
ASB1-Reverse:	5'-CTGGCAGAGATTGTATGTGAAGC-3'
PAT1-Forward:	5'-ATGGTTATTGCGGTGGCGACGA-3'
PAT1-Reverse:	5'-ATCGTCGCCGACTCAATGTCGG-3'
PAI1-Forward:	5'-CATCAGCCAGAGATGCAGCTA-3'
PAI1-Reverse:	5'-CAGAGGAATCAGCTGCTCTCA-3'
TSA1-Forward:	5'-CAGGCTGCGGCAACAAGGTCGT-3'
TSA1-Reverse:	5'-CAACAGCTCTGATGCTGGACAT-3'
TSB1-Forward:	5'-CTCACACGCACTAGCTTACCTC-3'

TSB1-Reverse:	5'-CATCAAGATATTTAGCCACTGT-3'
SUR2-Forward:	5'-CTCCCTTACCTAAAGGCAGTCA-3'
SUR2-Reverse:	5'-GCGTTCACCTGAATGATGGTCT-3'
SUR1-Forward:	5'-CGAGACCACCAAGGTGTTACAA-3'
SUR1-Reverse:	5'-TCAACATTATGTTTGAGTATCT-3'
NIT3-Forward:	5'-AGCGAAGTTGGTGTTGTTTCCC-3'
NIT3-Reverse:	5'-CCAACTCAGCCAATCTTTCCAC-3'
CYP79B2-Forward:	5'-CACGATGATGCTCGCGAGACT-3'
CYP79B2-Reverse:	5'-TCACTTCACCGTCGGGTAGAGA-3'
CYP79B3-Forward:	5'-CGTGATCCCTGTGACATGTCCT-3'
CYP79B3-Reverse:	5'-CACGCAGGTTTTGTAGCCGTTA-3'
PLT1-Forward:	5'-GAAGATGGCAAGCAAGGATCGG-3'
PLT1-Reverse:	5'-TCACGTCGTACCGGTTGATCTC-3'
PLT2-Forward:	5'-CAGACGCAGCTTCATCTTCACC-3'
PLT2-Reverse:	5'-ATCCAACGGTAGAGCTTGACCC-3'

Table S2. Genetic analysis of the *tkr1* mutant.

Cross (♀×♂)	Generation	Total seedlings tested	WT	TK VI-resistant[a]	χ^2
WT×*tkr1*	F1	20	0	20	/
WT×*tkr1*	F2	568	136	432	0.3380[b]

[a]TK VI-resistant phenotype was determined by primary root length after treated with 3 µM TK VI for 6 DAG.

[b]The calculated value was based on the expected ratio of TK VI-resistant seedlings over wild-type (WT) seedlings equals 3:1, P > 0.05.
